# Tailored Catalytic Microenvironments Enable Efficient Electrochemical Ammonia Production

**DOI:** 10.1002/advs.75265

**Published:** 2026-04-13

**Authors:** Qi Zhang, Peimiao Zou, Huimin Zhang, Alex J. Brown, Yingjie Song, Marc Walker, Yisong Han, Kui Xie, Shanwen Tao

**Affiliations:** ^1^ School of Engineering University of Warwick Coventry UK; ^2^ Photoemission Research Technology Platform and Department of Physics University of Warwick Coventry UK; ^3^ Electron Microscopy Research Technology Platform and Department of Physics University of Warwick Coventry UK; ^4^ School of Mechanical Engineering Shanghai Jiao Tong University Shanghai China

**Keywords:** catalytic microenvironment, decoupled adsorption–activation site, Lewis acid‐base, surface‐active H

## Abstract

Multi‐electron hydrogenation reactions are central to sustainable energy and nitrogen cycling but require catalysts with precise proton‐coupled electron transfer (PCET) and intermediate control. Here we report an integrated catalytic microenvironment (ICM) in a Co‐substituted inverse spinel oxide (FeFeCoO_4_) that enables efficient nitrate‐to‐ammonia reduction (NO_3_RR). Guided by crystal field and Lewis acid–base principles, Co^2^^+^ substitution induces Jahn–Teller distortions that activate otherwise inert tetrahedral Fe^3^^+^ sites as H* donors. Concurrently, hard–soft acid–base interactions establish decoupled dual‐metal sites: Co^2^^+^ stabilizes nitrogen intermediates, while Fe^3^^+^ forms Fe─O antibonding interactions that facilitate PCET. This ICM directs electron flow, promotes hydrogenation, and suppresses hydrogen evolution. Consequently, FeFeCoO_4_ achieves a peak NH_3_ yield of 1.89 × 10^−^^6^ mol s^−^^1^ cm^−^^2^ with 96.6% Faradaic efficiency and 30.5% energy efficiency, and stable operation at 0.5 A cm^−^^2^ for 265 h. This strategy offers a blueprint for catalysts for multi‐electron hydrogenations beyond nitrate reduction.

## Graphical abstract

This study shows that Co^2^^+^ substitution in inverse spinel FeFeCoO_4_ constructs an integrated catalytic microenvironment for efficient nitrate‐to‐ammonia reduction. Jahn–Teller distortion activates Fe sites for *H generation, while hard–soft acid–base regulated dual‐metal sites optimize proton‐coupled electron transfer and intermediate adsorption, delivering high Faradaic efficiency and ammonia yield.

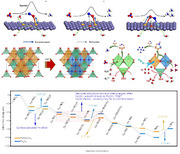

## 1 Introduction

Multi‐electron hydrogenation reactions, such as the reductions of N_2_, N_x_O_y_ to NH_3_ [1, 2, 3, 4, 5, 6, 7], and CO_2_ to fuels [8, 9, 10], are pivotal for sustainable energy systems, green fertilizer production, and restoring elemental cycles [11, 12]. These electrochemical processes convert abundant small molecules into value‐added products using renewable electricity [13]. Among them, electrochemical nitrate reduction to ammonia (NO_3_RR) is particularly attractive as it offers a sustainable alternative to the Haber–Bosch process, which consumes ∼1%–2% of global energy and emits large amounts of CO_2_ [13, 14, 15, 16], while simultaneously mitigating nitrate pollution [17]. However, like other multi‐electron hydrogenation reactions, NO_3_RR involves complex PCET steps and multiple intermediates [18, 19], which makes it difficult to achieve high selectivity. Recent advances highlight that the adsorption mode of N‐O intermediates plays a decisive role: promoting N‐O* adsorption over O─O* pathways enhances NH_3_ selectivity by suppressing hydrogen evolution [20, 21, 22]. Moreover, the hydrogenation of intermediates requires abundant surface H*, while HER competes for the same protons and electrons [23, 24]. Crucially, decoupling the adsorption site from the electron transfer site by separating where reactants or intermediates bind from where electron and proton delivery occurs enables directional electron flow, facilitates reaction pathway tuning by selectively stabilizing or accelerating desired intermediate transitions, and avoids site poisoning. Therefore, constructing integrated catalytic microenvironments that spatially co‐locate H* generation domains with neighboring electron‐rich centers for directional intermediate activation is essential. Such tandem or multifunctional systems improve PCET efficiency and product selectivity by orchestrating active hydrogen supply and oriented binding/activation of intermediates. Such designs are therefore critical for overcoming key bottlenecks in NO_3_RR and other multi‐electron hydrogenation reactions.

The key challenge lies in precisely designing such reaction sites for efficient multi‐electron nitrate reduction. Here, based on crystal field theory and Lewis acid‐base principles [25, 26, 27], we modulated the d‐band center and t_2g_ electrons of tetrahedral sites in the Co_x_Fe_3‐x_O_4_ spinel structure, successfully activating them as surface H* adsorption centers. Guided by the Hard soft acids bases (HSAB) principle [27], we constructed a dual‐metal‐site system featuring decoupled adsorption and activation functions: Octahedral Co sites, as moderate Lewis acids, favor nitrogen coordination, forming stable Co─N bonds that stabilize intermediates (*NO_2_ and *NO), while octahedral Fe sites, acting as strong Lewis acids, preferentially bind hard‐base oxygen to form Fe─O bonds with antibonding properties that enable directional electron flow and promote N─O bond cleavage and protonation. The resulting FeFeCoO_4_ catalyst exhibits outstanding NO_3_^−^RR performance, achieving >98% Faradaic efficiency (FE) across a wide potential range (−0.1 to –0.6 V vs. RHE), a high energy efficiency (EE) of 48.4%, and a peak NH_3_ yield rate of 1.89 × 10^−^^6^ mol s^−^^1^ cm^−^^2^ with 96.6% FE and 30.5% EE, which is far surpassing N_2_RR (∼1%). It also shows remarkable stability at 0.5 A cm^−^^2^ for 265 h with a degradation rate of only 0.25 mV h^−^^1^ and, long‐term durability in Zn─NO_3_^−^ batteries at 1 mA cm^−^^2^ for 500 h. The superior robustness is attributed to contract stress that stabilizes both active sites and the surface‐H sites. This strategy can be extended to other multi‐electron hydrogenation reactions.

## 2 Results and Discussion

### 2.1 Integrated Catalytic Microenvironments Design for the NO_3_RR

**FIGURE 1:**
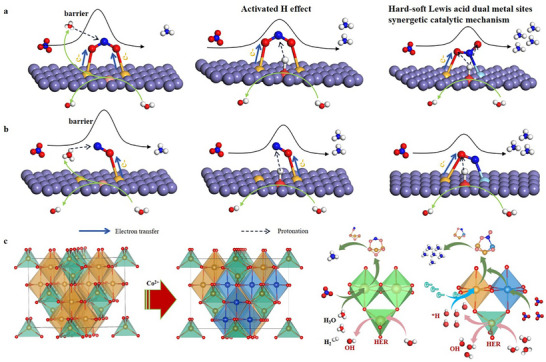
Integrated catalytic microenvironments design for the NO_3_RR. (a,b) Integration of activated hydrogen sites with hard–soft acid dual‐metal sites induces a favourable N─O adsorption configuration and decouples the adsorption and activation centers. (c) Schematic illustrating the enhanced catalytic mechanism of FeFeCoO_4_, originating from the H effect and decoupled adsorption/activation sites that optimize intermediate adsorption and promote the PCET process.

Figure 1a,b illustrates our proposed catalytic microenvironment with three functionally orthogonal sites: (i) *H generation, (ii) intermediates (*NO_2_ and *NO) adsorption, and (iii) decoupled electron‐rich activation, the latter enabling reduction catalysis while cooperatively enhancing adsorption. In theory, such integration can be achieved using multi‐atomic site catalysts. However, in practice, this type of microenvironment tends to form randomly and uncontrollably within these systems (Figure S1).

Among various candidates, the inverse spinel structure with its unique combination of tetrahedral (Td) and octahedral (Oh) coordination environments emerges as one of the most promising platforms for constructing integrated microenvironments [28]. On one hand, crystal field theory suggests that different metal ions, or even different oxidation states of the same ion, preferentially occupy specific coordination sites (Td vs. Oh) [29]. On the other hand, in inverse spinel oxide, a tetrahedral center is bridged by two adjacent octahedral centers, forming a triangular configuration. In this geometry, the two more catalytically active octahedral metal centers possess e_g_ orbitals oriented directly toward ligands, enabling strong σ‐bonding and high degrees of orbital hybridization with ligand p orbitals (e.g., O 2p), thus making them ideal candidates for adsorption‐activation sites. In contrast, the tetrahedral metal center, with t_2g_ orbitals directed between ligands and weaker hybridization, can act as a relatively inert but stable site for *H generation. Spinel catalysts such as Fe_3_O_4_ and its derivatives have been reported as potential NO_3_RR catalysts [30, 31, 32]. However, these typically suffer from inactive tetrahedral Fe sites, leading to a significant deficiency in H* generation and suboptimal NH_3_ production. Furthermore, Fe_3_O_4_ has been shown to preferentially promote O─O adsorption configurations, which are unfavorable for ammonia synthesis [33].

As shown in Figure 1c, to address these issues, we propose substituting octahedral Fe^2^^+^ with Co^2^^+^ to induce a Jahn–Teller effect, thereby activating adjacent tetrahedral centers as *H generation sites. Moreover, based on the HSAB principle and Lewis's acid‐base theory, Fe^3^^+^ (Oh), as a hard acid, tends to bind strongly with hard bases such as O, whereas Co^2^^+^ (Oh), being a moderate Lewis acid, shows stronger affinity toward softer bases like N. This substitution allows us to modulate the adsorption configuration of key NO_3_RR intermediates by shifting from unfavorable O─O adsorption to a more favorable N─O adsorption, thereby enhancing the Faradaic efficiency for ammonia production. In this way, the envisioned integrated catalytic microenvironment can be constructed to improve the NH_3_ yield rate and FE for the NO_3_RR.

### 2.2 Materials Synthesis and Characterization

**FIGURE 2:**
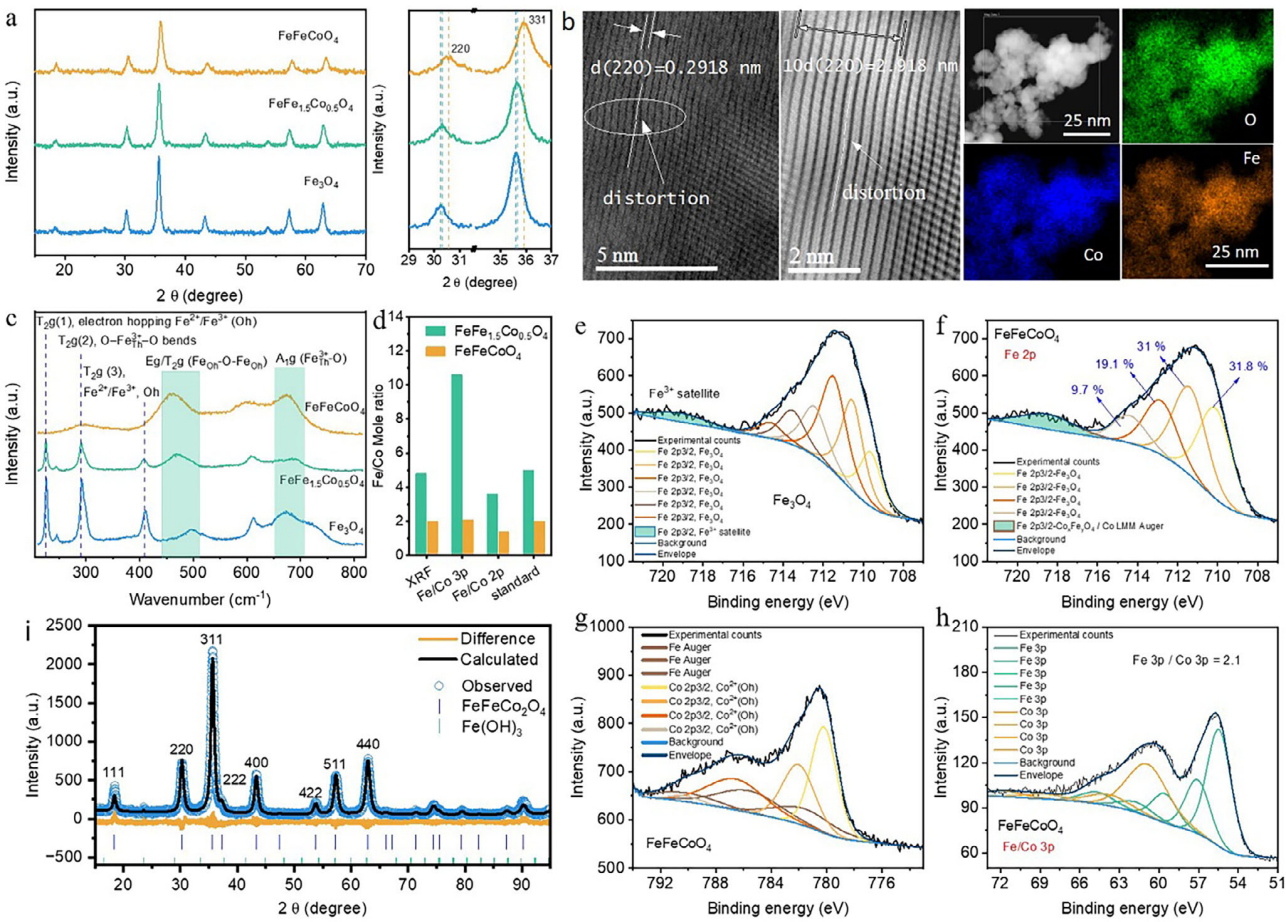
Structural characterization of Co_x_Fe_3‐x_O_4_. (a) XRD patterns. (b) Bright‐field and annular dark‐field STEM images of FeFeCoO_4_ and corresponding EDX elemental maps. (c) Raman spectra. (d) Metal element atomic ratio of Fe/Co. (e–h) Fe 2p XPS for Fe_3_O_4_ (e) and FeFeCoO_4_ (f), Co 2p and 3p XPS for FeFeCoO_4_ (g). (i) Rietveld refinement of FeFeCoO_4_.

As schematically illustrated in Figure S2 Co_x_Fe_3‐x_O_4_ (x = 0,0.5, 1) catalysts were synthesized through a co‐deposition precipitation and a following thermal decomposition [34]. XRD patterns (Figure 2a) confirm that Co‐doped Fe_3_O_4_ catalysts retain a spinel structure, with diffraction peaks gradually shifting to higher angles upon increasing Co content. This indicates lattice contraction, attributed to the smaller ionic radius of Co^2^^+^ compared to Fe^2^^+^, and the resulting strain‐induced Jahn‐Teller distortion [35, 36]. High‐resolution scanning transmission electron microscopy (STEM) images (Figure 2b) further support this, showing a reduced interplanar spacing of 0.2918 nm for the (220) plane and local lattice distortions [37]. The EDX elemental maps reveal that the Fe, Co, and O elements are homogeneously distributed in the Co_x_Fe_3‐x_O_4_ [37] (Figure 2b and Figures S3–S5). Figure S6, the scanning electron microscopy (SEM) images display their morphology structure. Raman spectra (Figure 2c) reveal that Co substitution disrupts vibrational modes associated with the spinel lattice. Specifically, the ∼200 cm^−^^1^ (T_2g_, electron hopping Fe^2+^/Fe^3+^), ∼245 cm^−^^1^ (T_2g_, tetrahedral O‐bending) and 410 cm^−^^1^ (T_2g_, B‐site sensitive) modes vanish in FeFeCoO_4_ due to strong Jahn‐Teller effects and local octahedral‐site disorder [38], which demonstrated the fully substitution of Fe^2+^(Oh) by Co(Oh) for the FeFeCoO_4_. These distortions break local symmetry and violate Raman selection rules, confirming the destabilization of Fe‐O vibrations. Moreover, Co^2^^+^ incorporation suppresses spin‐phonon coupling by freezing electron hopping (Fe^2^^+^ ↔ Fe^3^^+^), in contrast to Fe_3_O_4_ where dynamic charge transfer results in sharp phonon features. The absence of the 310 cm^−^^1^ mode and broadening of all phonon bands further indicate mass disorder, lattice strain, and cation redistribution [38].

X‐ray photoelectron spectroscopy (XPS) was conducted to identify the metal coordination environment (Figure 2e–h, Figure S7). As shown in Figure 2d, XPS and XRF reveal Co surface segregation, with a surface Co/Fe atomic ratio of 1.4 (XPS 2p) vs. a bulk ratio of 2.1 (XPS 3p). As shown in Figure 2e,f, the Fe 2p spectrum of FeFeCoO_4_ resembles that of NiFe_2_O_4_ [39], indicating Fe element with +3 and a comparable chemical environment, further supported by the Co 2p spectra. Co^2^^+^ is identified as the dominant bulk species (Figure 2g), consistent with an inverse spinel structure [Fe^3^^+^]_Td_[Co^2^^+^Fe^3^^+^]_Oh_O_4_ [40]. Minor Fe(OH)_3_ phases were detected by XRD refinement (Figure 2i), and O 1s XPS (Figure S7) indicates surface hydroxide and adsorbed nitrate species.

### 2.3 The Activation Mechanism of Fe (Td) as Surface‐active H Sites

**FIGURE 3:**
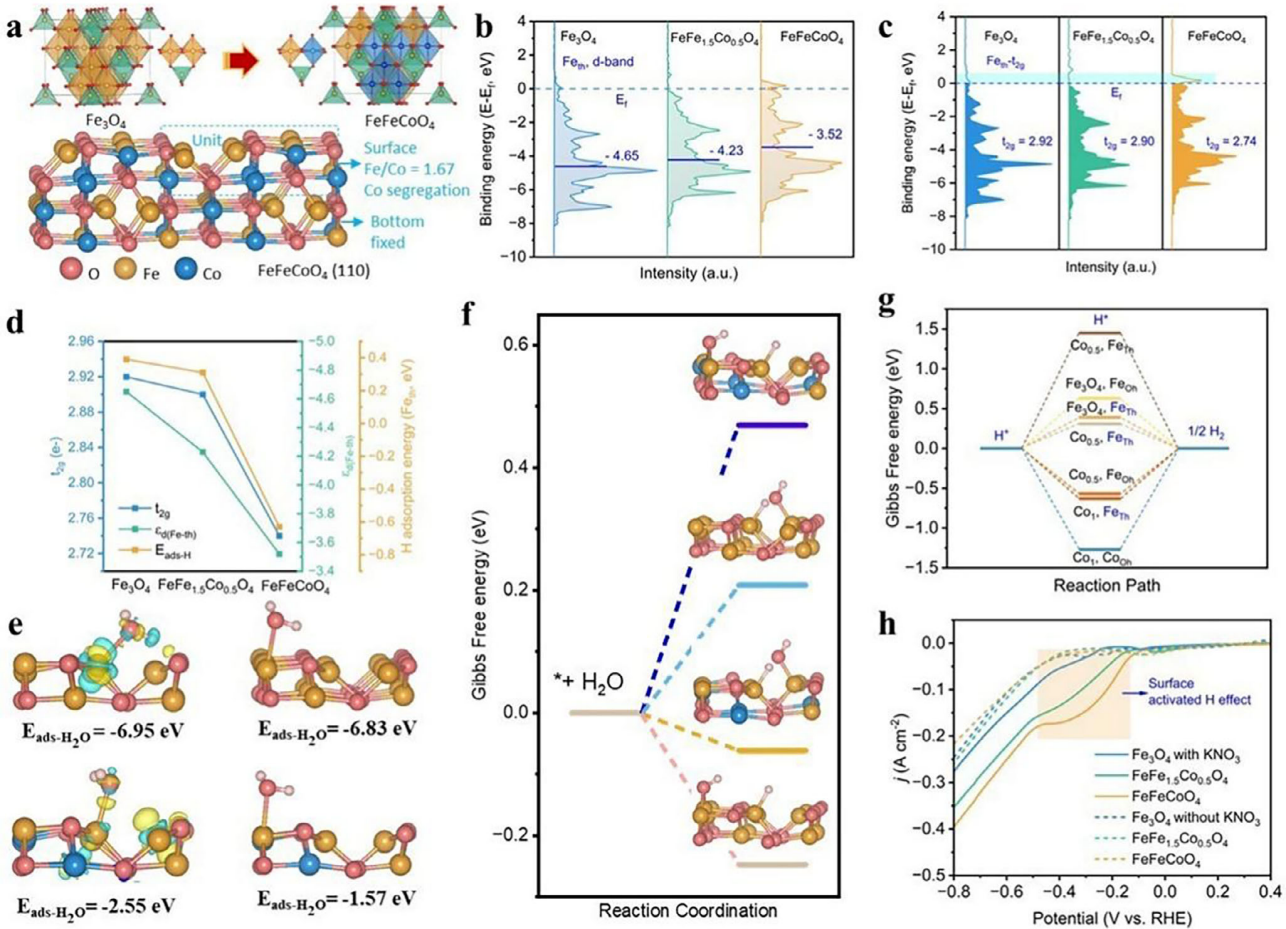
Activation mechanism of Fe (Td) as surface‐active H sites. (a) Simulated model for FeFeCoO_4_ (110). d‐band electronic state density (b) and t_2g_ orbitals (c) analysis and corresponding relationship with H adsorption energy (d). (e) Adsorption models of H_2_O on FeFeCoO_4_ (110) and Fe_3_O_4_ (110), with corresponding adsorption energy. (f) Gibbs free energy diagrams for H_2_O dissociation on FeFeCoO_4_ (110) and Fe_3_O_4_ (110), respectively. (g) Gibbs free energy diagrams of the HER on Fe_3_O_4_ (110), FeFe_1.5_Co_0.5_O_4_ (110), and FeFeCoO_4_ (110). (h) LSV curves for NO_3_RR over the as‐prepared Co_x_Fe_3‐x_O_4_ in 1 M KOH + 0.1 M KNO_3_ electrolyte (loading mass: 2 mg cm^−2^).

Based on the characterization results, a corresponding model was constructed (Figure 3a) to investigate the electronic structure of the integrated catalytic microenvironment (ICM). Density of states (DOS) calculations (Figure 3b) reveal that increasing Co content shifts the d‐band center of Fe (Td) closer to the Fermi level from – 4.65 eV in Fe_3_O_4_ to –3.52 eV in FeFeCoO_4_, indicating enhanced hydrogen adsorption strength (Figure 3d). Further analysis of the t_2g_ orbitals (Figure 3c) shows lower electron occupancy in FeFeCoO_4_, suggesting stronger orbital hybridization with hydrogen. Additionally, FeFeCoO_4_ exhibits a higher electron state density near the Fermi level than Fe_3_O_4_, which facilitates PECT during catalysis. As shown in Figure 3e, FeFeCoO_4_ (110) exhibits a markedly stronger H_2_O adsorption at tetrahedral sites (−2.55 eV) than at octahedral sites (−1.57 eV), reflecting a clear site selectivity. In contrast, Fe_3_O_4_ (110) shows comparable adsorption energies at both sites, while the stronger H_2_O adsorption relative to NO_3_^−^ (Figure S8) severely suppresses NO_3_^−^ reduction. Gibbs free energy profiles (Figure 3f) reveal favourable H_2_O dissociation on both surfaces (−0.25 eV for FeFeCoO_4_ (110) and –0.06 eV for Fe_3_O_4_ (110)), indicative of intrinsically fast kinetics. Crucially, the dissociation products adopt distinct configurations: on FeFeCoO_4_ (110), OH and H preferentially occupy tetrahedral sites, accelerating protonation, whereas on Fe_3_O_4_ (110), OH anchors at octahedral sites, restricting NO_3_^−^ adsorption and reduction. Moreover, the *H free energy diagrams (Figure 3g) demonstrate that Co substitution not only activates otherwise inert Fe (Td) sites for H adsorption but also enhances the hydrogen affinity of Fe (Oh) sites, thereby optimizing the surface for NO_3_^−^ reduction. On FeFeCoO_4_ (110), all exposed surface metal sites become active *H adsorption sites, while Fe_3_O_4_ (110) shows none. These theoretical insights align with experimental LSV measurements in 1 M KOH, with 0.1 M KNO_3_. Upon nitrate addition, all catalysts show increased current density (Figure 3h), indicating ongoing NO_3_^−^RR. Notably, FeFeCoO_4_ exhibits three distinct stages: (I) a rapid current rise (0 to –0.25 V vs. RHE) due to abundant *H adsorption; (II) a slower rise (−0.25 to –0.5 V) caused by limited *NO_3_ availability; and (III) a sharp increase beyond –0.5 V due to accelerated *H generation via HER and enhanced reaction kinetics of NO_3_RR under relatively negative applied potential. The distinct three‐stage current response reflects the transition from mass transport‐limited NO_3_^−^ reduction to kinetically controlled NO_3_RR with increasing nitrate availability.

These findings confirm that Co incorporation activates Fe sites and enhances both NO_3_^−^RR and *H effect through optimized electronic structure and surface reactivity.

### 2.4 Mechanism of Decoupled Adsorption–Activation Site for NO_3_RR

**FIGURE 4:**
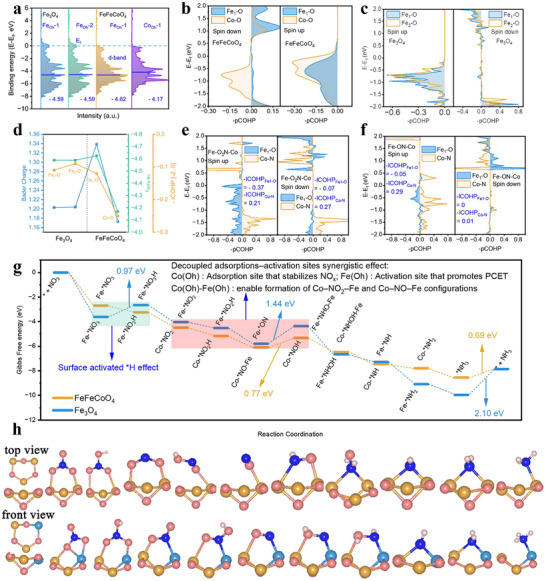
Mechanism of decoupled adsorption–activation site for NO_3_RR. (a) d‐band center calculation for the octahedral sites of the ICM on Fe_3_O_4_ (110) FeFeCoO_4_ (110). (b,c) Crystal Orbital Hamilton Population (COHP) analysis to investigate the bond properties of Fe─O and Co─O of the ICM on Fe_3_O_4_ (110) and FeFeCoO_4_ (110). (d) Bader charge analysis, d‐band center Co_Oh_ and Fe_Oh_, with corresponding boning/antibonding strengths of Fe/Co─O bonds. (e,f) COHP analysis of Fe─O and Co─N interactions in *NO_2_ and *NO intermediates on FeFeCoO_4_ (110), integrated over the interval [‐2, 0] for ‐ICOHP calculations. (g) Gibbs free energy diagrams of NO_3_RR for Fe_3_O_4_ (110) and FeFeCoO_4_ (110). (h) Optimized intermediate structures.

We further explored the Lewis acid‐base properties of the octahedral sites of the ICM to elucidate the role of Fe_Oh_‐Co_Oh_ in decoupling adsorption and activation functionalities during nitrate reduction (NO_3_RR). As shown in Figure 4a, d‐band center calculations show that Co_Oh_ atom exhibits a d‐band center closer to the Fermi level than Fe_Oh_ atom, suggesting a higher electronic activity. Further COHP analysis (Figure 4b) supports this distinction: Fe─O bonds are dominated by bonding states with minimal antibonding contributions near the Fermi level, typical of hard Lewis acids. In contrast, Co─O bonds exhibit pronounced antibonding states near the Fermi level (Figure 4c), indicating lower electron affinity and confirming Co_Oh_ as a moderate Lewis acid site. The integrated crystal orbital Hamilton population (ICOHP) values confirm that Co_Oh_‐O bonds are weaker than Fe_Oh_─O bonds (Figure 4d), and Bader charge analysis (Figure 4d) reveals a lower positive charge on Co_Oh_. These results classify Co_Oh_ as a moderate Lewis acid site, in contrast to Fe_Oh_, which behaves as a strong Lewis acid site due to its stronger metal‐oxygen bonding and greater electron‐accepting ability. This dual‐site environment with hard Fe_Oh_ and moderate Co_Oh_ enables a unique Co─N─O─Fe adsorption configuration for NO_x_ intermediates, facilitating selective NH_3_ generation. COHP analysis provides critical insights into the electronic origin of the enhanced NO_3_RR activity on FeFeCoO_4_. For the key intermediates *NO_x_ (Figure 4e) and *NO (Figure 4f), the Co─N bond exhibits strong bonding states near the Fermi level, demonstrating that Co(Oh) sites act as moderate Lewis acids that optimize NO_x_ adsorption. This leads to the formation of a Fe─O─N─Co bridging configuration, consistent with the Gibbs free energy results (Figure S9), thereby stabilizing reaction intermediates. In parallel, the Fe─O bond, with O derived from NO_x_, shows pronounced antibonding states near the Fermi level, signifying enhanced reactivity and promoting proton‐coupled electron transfer (PCET). This electronic modulation establishes a cooperative division of labor: Co(Oh) sites refine adsorption, while Fe centers accelerate bond activation and PCET.

Gibbs free energy calculations further illustrate how this synergy translates into reaction kinetics (Figure 4g,h, and Figures S10 and S11). On Fe_3_O_4_, the reaction pathway is hindered by substantial barriers for *NO_3_ protonation (0.97 eV) and *NO protonation (1.44 eV), due to limited *H availability and suboptimal Fe─ON geometry. In sharp contrast, FeFeCoO_4_ exhibits an energetically favorable landscape: *NO_3_ protonation becomes exothermic, and the barrier for *NO protonation is reduced to 0.77 eV. Although the *NO → *NOH step remains the rate‐determining step, the lowered barrier markedly accelerates the process. These improvements arise from two intertwined factors: (i) enhanced *H formation enabled by the activation of Fe(Td) and Fe(Oh) sites, and (ii) a synergistic adsorption–activation mechanism, wherein Co(Oh) drives the Fe─O─N─Co adsorption motif while Fe(Oh) promotes PCET. Together, these features establish an electronic and structural environment that lowers energy barriers, accelerates PCET kinetics, and directs the pathway toward selective NH_3_ production. This mechanistic framework underscores the broader design principle that ICM can strategically tailor site‐specific electronic structures to balance intermediate stabilization with facile charge transfer, thereby enabling efficient NO_3_RR catalysis

### 2.5 The Electrocatalytic Nitrate Reduction (NO_3_RR) Performance of FeFeCoO_4_

**FIGURE 5:**
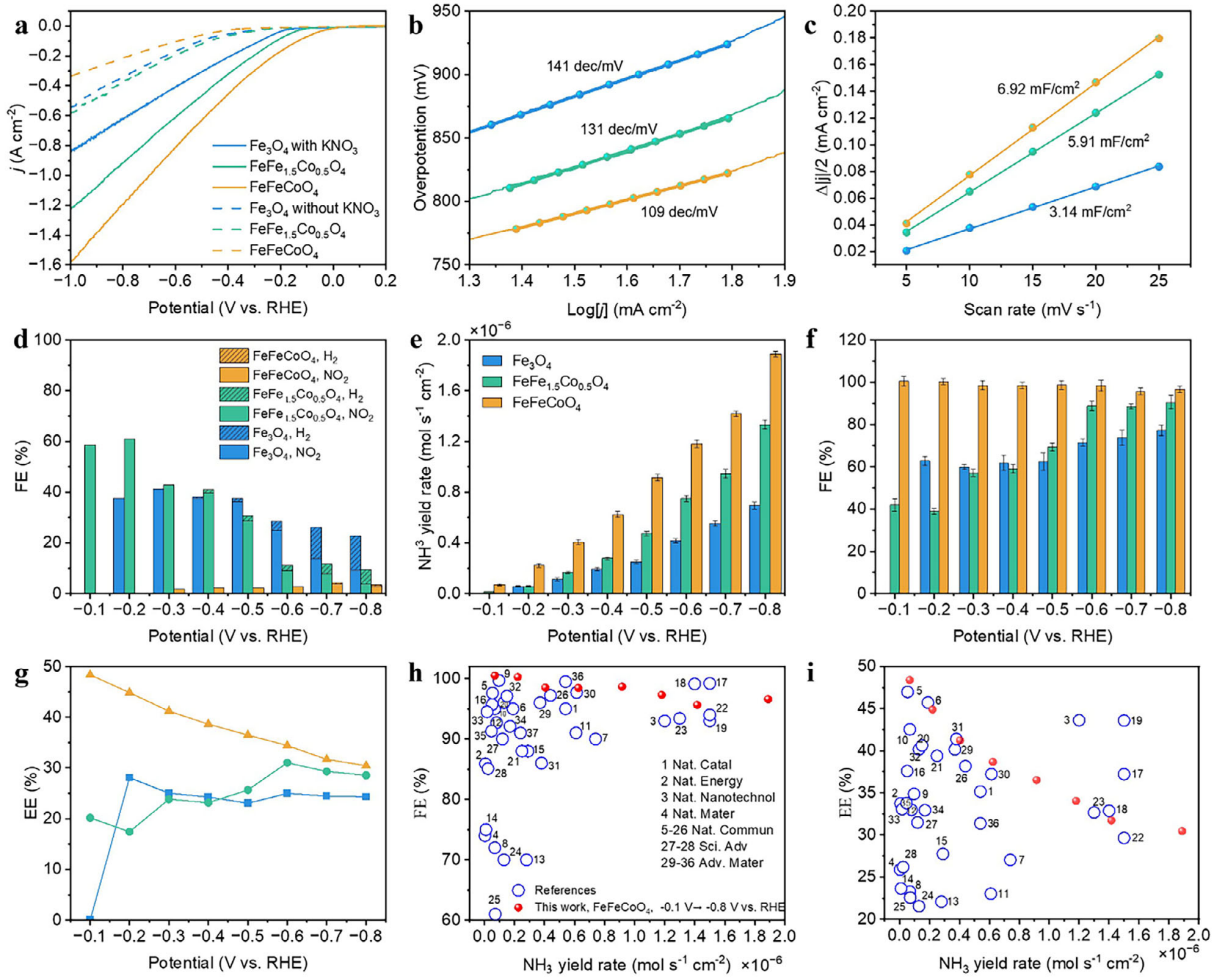
NO_3_RR performance of Co_x_Fe_3‐x_O_4_. LSV curves (a) and Tafel plots (b) over Co_x_Fe_3‐x_O_4_ at a scan rate of 10 mV/s in 1 M KOH + 1 M KNO_3_ electrolyte. (c) ECSA analysis by C_dl_ method. (d) Faradic efficiency of byproducts NO_2_^−^ and H_2_. NH_3_ yield rate (e), faradic efficiency (f), and energy efficiency (g) of Co_x_Fe_3‐x_O_4_ under different applied potentials. Comparisons of NO_3_RR performances in terms of the faradic efficiency (h) and energy efficiency (i) vs. NH_3_ yield rate between FeFeCoO_4_ and recent reported catalysts (for details, see Table S1).

The electrocatalytic nitrate reduction (NO_3_RR) performance of FeFeCoO_4_ was systematically evaluated in 1 M KOH electrolyte with and without added nitrate (0.1 M and 1 M KNO_3_), using linear sweep voltammetry (LSV) at a scan rate of 10 mV s^−^^1^ over a potential range of 0.4 V to −1.0 V vs. RHE at ambient temperature. As shown in Figure 5a, FeFeCoO_4_ demonstrates significantly enhanced NO_3_RR activity, characterized by a low onset potential of −0.1 V and a high industrial‐level current density of 1.6 A cm^−^^2^ at −1.0 V vs. RHE. As shown in Figure 5b, the Tafel slope of FeFeCoO_4_ is 109 mV dec^−^^1^, much lower than that of Fe_3_O_4_ (141 mV dec^−^^1^), indicating more favorable kinetics and faster electron‐transfer rates for NO_3_RR. Electrochemical surface area (ECSA), estimated via double‐layer capacitance (C_dl_) measurements (Figure 5b), further confirms that FeFeCoO_4_ possesses the largest electrochemically active area with the corresponding ECSA of 173 cm^2^ among the tested catalysts, including Fe_3_O_4_ and FeFe_1_._5_Co_0_._5_O_4_ (Figure S12), suggesting a greater density of exposed active sites. As shown in Figures S13,  S14, and Tables S2,  S3, ECSA‐normalized and A_BET_‐normalized current density and NH_3_ yield rate demonstrate that the catalytic improvement is not governed by the total physical surface area measured by Brunauer–Emmett–Teller (BET) method, but by the synergistic optimization of active‐site accessibility and intrinsic catalytic properties, enabled by the tailored Fe‐Co dual‐site catalytic microenvironment.

Quantitative analyses of reaction products were carried out by UV–vis colorimetry (Figures S15 and S16) after chronoamperometry tests in different potentials (Figures S17–S20). These results reveal that with increasing Co content, the competing hydrogen evolution reaction (HER) is significantly suppressed, and the formation of the undesired nitrite byproduct (NO_2_^−^) is minimized (Figure 5d).

As shown in Figure 5e, among the tested compositions, FeFeCoO_4_ exhibits the best overall NO_3_^−^RR performance, achieving a high FE exceeding 99% over a wide potential range (−0.1 to −0.6 V vs. RHE). Notably, at −0.8 V, FeFeCoO_4_ delivers an NH_3_ yield rate of 1.89 × 10^−^^6^ mol s^−^^1^ cm^−^^2^ with an FE of 97%, surpassing the U.S. Department of Energy (DOE) target for electrochemical ammonia synthesis from N_2_ (9.73 × 10^−^^7^ mol s^−^^1^ cm^−^^2^ and 90% FE) [41]. Energy efficiency (EE) calculations (Figure 5e) further confirm the practical viability of FeFeCoO_4_: an EE of 48% is achieved at −0.1 V vs. RHE, which is close to the Haber–Bosch process (55%–68%) [42]. These results collectively highlight FeFeCoO_4_ as a highly promising catalyst for electrochemical ammonia production, outperforming many state‐of‐the‐art NO_3_^−^RR catalysts in terms of FE, yield rate, and EE over a broad operating window(Figure 5f,g and Table S1) [43, 44, 45, 46, 47, 48, 49, 50, 51].

**FIGURE 6:**
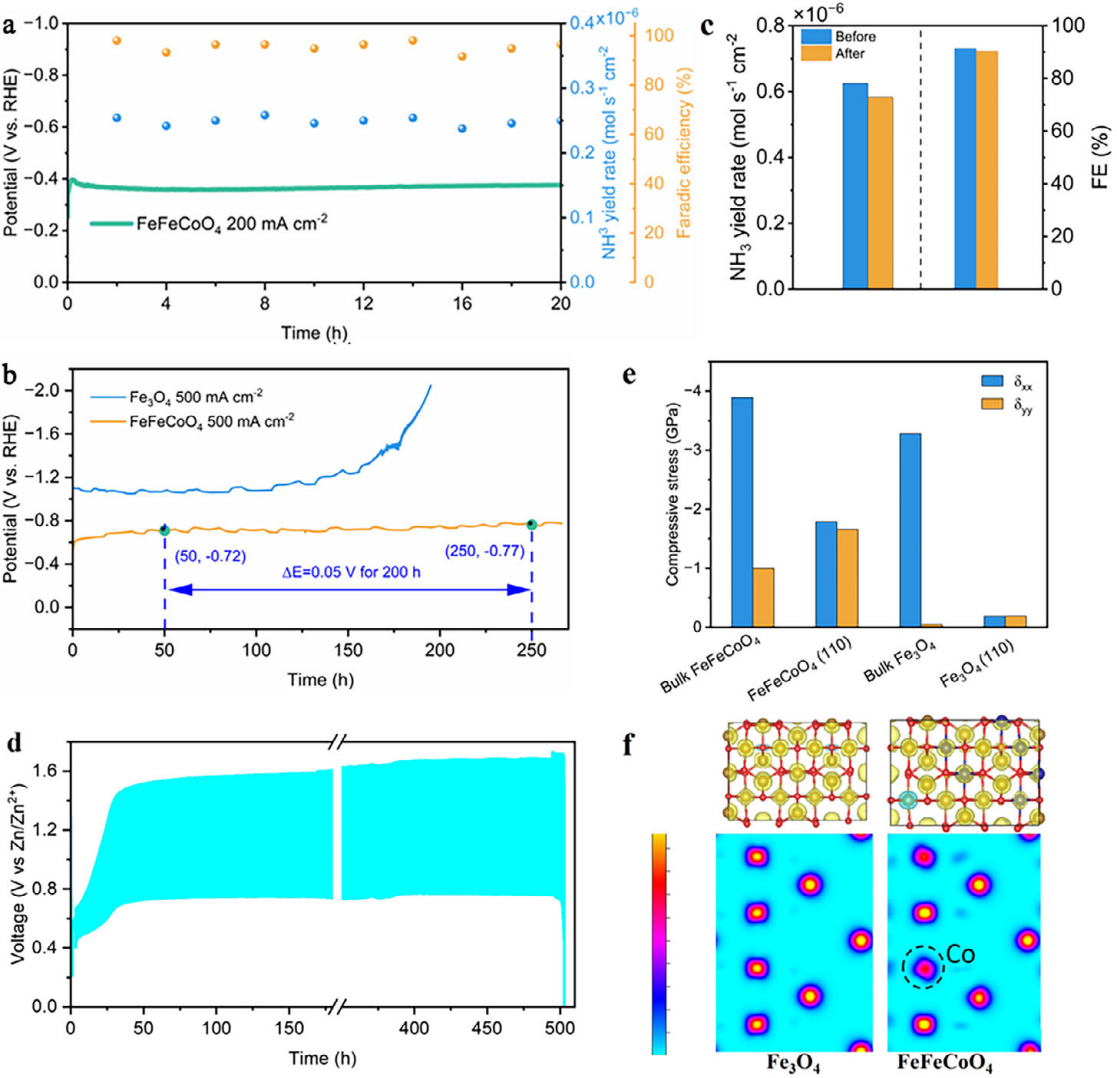
Stability of Co_x_Fe_3‐x_O_4_ for NO_3_RR and Zn‐NO_3_ battery. (a) Chronopotentiometry curve of FeFeCoO_4_ in an H‐type continuous‐flow cell at 200 mA cm^−2^ with corresponding Y_NH3_ and FE_NH3_. (b) Long‐term stability for NO_3_RR over Fe_3_O_4_ and FeFeCoO_4_ (1 M KOH + 0.1 M KNO_3_ electrolyte, 0.5 A cm^−2^) and the comparison of the Y_NH3_ and FE_NH3_ before and after testing (c). (d) The discharge‐charge profile for Zn‐NO_3_ battery in 3 M KOH + 0.5 M KNO_3_ cathodic electrolyte and 3 M KOH + 0.2 M ZnAc_2_ anodic electrolyte at 1 mA cm^−2^. (e), The stress of bulk Fe_3_O_4_ and FeFeCoO_4,_ Fe_3_O_4_ (110) and FeFeCoO_4_ (110). (f), Surface spin state of Fe_3_O_4_ and FeFeCoO_4_.

To evaluate the catalyst's durability, FeFeCoO_4_ was subjected to long‐term electrolysis in 1 M KOH + 0.1 M KNO_3_, simulating real nitrate‐contaminated wastewater conditions with a low concentration of NO_3_^−^. Compared to Fe_3_O_4_ and FeFe_1_._5_Co_0_._5_O_4_, FeFeCoO_4_ maintains superior FE and NH_3_ yield (Figures S21–S25). In a 20‐h continuous electrolysis at −0.4 V vs. RHE in an H‐cell (Figure 6a), FeFeCoO_4_ displays minimal fluctuations in NH_3_ yield (∼2.5 × 10^−^^7^ mol s^−^^1^ cm^−^^2^) and FE (∼96%), confirming its robust electrochemical stability. Post‐reaction characterizations (Figure S26) confirm that the spinel crystal structure remains intact.

For further validation, FeFeCoO_4_ was tested under industrially relevant conditions using chronopotentiometry at 500 mA cm^−^^2^ under continuous flow. As shown in Figure 6b,c, the catalyst exhibits remarkable long‐term stability for over 265 h, maintaining both NH_3_ yield and FE with a low voltage degradation rate of just 0.25 mV h^−^^1^. In comparison, Fe_3_O_4_ shows a significant voltage increase after 150 h. Moreover, a Zn–NO_3_ battery assembled using FeFeCoO_4_ as the cathode also delivers excellent performance over 500 h at 1 mA cm^−^^2^ in 3 M KOH + 0.5 M KNO_3_ (Figure 6d), further demonstrating its practical applicability.

The outstanding catalytic stability of FeFeCoO_4_ is attributed to its integrated catalytic microenvironment, consisting of activated Fe_Td_ sites for *H generation and spatially separated Co_Oh_‐Fe_Oh_ decoupled adsorption–activation centers. Additionally, mechanical simulations suggest that FeFeCoO_4_ exhibits compressive lattice stress, which contributes to the stabilization of interfacial cation migration (ICM) sites, enhancing the structural integrity and catalytic durability of the electrode under prolonged operation. Surface spin state analysis (Figure 6f) indicates that the FeFeCoO_4_ possesses a lower spin charge compared with Fe_3_O_4_, implying a better structure stability. The findings firmly establish FeFeCoO_4_ as a highly efficient, stable, and industrially applicable electrocatalyst for nitrate‐to‐ammonia conversion.

## 3 Discussion

In summary, we report a strategically integrated catalytic microenvironment within inverse spinel FeFeCoO_4_ to overcome efficiency and selectivity bottlenecks in the nitrate reduction reaction (NO_3_RR). Guided by crystal field theory and hard‐soft acid‐base principles, cobalt substitution induces a Jahn‐Teller distortion that activates inert tetrahedral Fe sites (Fe_Td_) for efficient surface‐active hydrogen (H*) generation. This architecture creates spatially decoupled adsorption–activation sites: Co_Oh_ moderates N coordination to stabilize NO_x_ intermediates, while Fe_Oh_ strengthens Fe─O interactions with antibonding character, serving as electron‐rich centers that drive directional transfer and accelerate N─O bond cleavage. Synergistically, the integration of H* supply from Fe_Td_, oxygen binding at Fe_Oh_, and nitrogen stabilization at Co_Oh_ optimizes proton‐coupled electron transfer and suppresses competing pathways. The catalyst achieves >98% NH_3_ Faradaic efficiency (−0.1 to –0.6 V vs. RHE), a peak yield of 1.89 × 10^−^^6^ mol s^−^^1^ cm^−^^2^with 96.6% FE and 48.4% EE. It demonstrates exceptional stability at a high current density of 0.5 A cm^−^^2^ for 265 h with degradation of only 0.25 mV h^−^^1^ due to a stabilized microenvironment from compressive lattice stress. This site‐decoupling strategy provides a general design framework for multi‐electron hydrogenation reactions.

## 4 Experimental Section/Methods

All experimental details are included in the Supporting Information.

## Conflicts of Interest

The authors declare no conflicts of interest.

## Supporting information




**Supporting File**: advs75265‐sup‐0001‐SuppMat.pdf.

## Data Availability

The data that support the findings of this study are available on request from the corresponding author. The data are not publicly available due to privacy or ethical restrictions.
