# Supplementary material for: Tailored Catalytic Microenvironments Enable Efficient Electrochemical Ammonia Production
Source: Adv Sci (Weinh). 2026 Apr 13;13(44):e75265. doi: 10.1002/advs.75265 (PMC13334945; doi:10.1002/advs.75265)
Supplement: Supplementary file 1 [file ADVS-13-e75265-s001.pdf]

Tailored Catalytic Microenvironments Enable Efficient Electrochemical Ammonia Production

*Qi Zhang, Peimiao Zou, Huimin Zhang, Alex J. Brown, Yingjie Song, Walker, Marc, Yisong Han, Kui Xie, Shanwen Tao**

Q. Zhang, P. Zou, H. Zhang, A. Brown, Y. Song, S. Tao
School of Engineering, University of Warwick, Coventry CV4 7AL, UK

E-mail: S.Tao.1@warwick.ac.uk

W. Marc
Photoemission Research Technology Platform and Department of Physics, University of Warwick, Coventry CV4 7AL, UK
Y. Han
Electron Microscopy Research Technology Platform and Department of Physics, University of Warwick, Coventry, CV4 7AL, UK
K. Xie
School of Mechanical Engineering, Shanghai Jiao Tong University, 800 Dongchuan Road, Shanghai 200240, China

Keywords: catalytic microenvironment; Lewis acid-base; surface-active H; decoupled adsorption–activation site

Table of Contents

Supplementary Text

Figures S1-S26

Tables S3

Supplementary Text

Methods

Synthesis of the $Co_xFe_{3-x}O_4$ catalysts

Fe_3O_4, $FeFe_{1.5}Co_{0.5}O_4$ and $FeFeCoO_4$ nanoparticles were all prepared by a co-deposition precipitation and a following thermal decomposition. 0.02 mol Iron (III) nitrate nonahydrate, 98+% (metals basis), and 0.01 mol Cobalt (II) nitrate hexahydrate were completely dissolved in 50 ml deionized water. Subsequently, 150 ml KOH (1 M) was added under continuous N_2 and with continuously stirring under room temperature. The precipitate was washed with hot deionized water (60 °C) for several times and then collected by centrifugation and dry at 130 °C for 24 h. Finally, the black power of $FeFeCoO_4$ was obtained after a following calcined at 350 °C in air for 24 h. The Fe_3O_4 and $FeFe_{1.5}Co_{0.5}O_4$ were also prepared with the same method with corresponding metal salt. For synthesis of Fe_3O_4, 0.02 mol Iron (III) nitrate nonahydrate, 98+% (metals basis), and 0.01 mol Iron (II) sulphate heptahydrate 99% were used. 0.02 mol Iron (III) nitrate nonahydrate, 98+% (metals basis), 0.005 mol Cobalt (II) nitrate hexahydrate and 0.01 mol Iron (II) sulphate heptahydrate 99% were used for the synthesis of $FeFe_{1.5}Co_{0.5}O_4$.

Materials characterization

The X-ray diffraction (XRD) with a Cu Kα radiation (1.5419 Å) was performed on to identify the crystalline phases. The diffraction patterns were collected over a 2θ range from 5° to 90° at a step size of 0.013° with a counting time of 110 s per step and were analyzed using the Malvern Panalytical Highscore Plus 4.9 software and the latest ICDD PDF-4+ database. The high-quality XRD data was collected by extending the scanning time to 6 h for refinement. Rietveld refinement of the objective spinel oxide $FeFeCoO_4$ was carried out by GSAS-II75.

Scanning electron microscopy (SEM) equipped with Energy dispersive X-ray spectroscopy (EDX) was conducted to investigate the morphology of the catalyst and the corresponding element distribution. A double aberration-corrected JEOL ARM200F TEM equipped with a

100 mm^2 Oxford Instruments, operated at 200 kV, was used to observe the structure of the catalyst and to analyze its elemental distribution.

The x-ray photoelectron spectroscopy (XPS) data were collected at the Photoemission Research Technology Platform, University of Warwick. The samples investigated in this study were attached to electrically conductive carbon tape and mounted on to a sample bar with a layer of filter paper separating the sample bar and the carbon tape to ensure electrical isolation and hence eliminate the possibility of differential charging due to poor electrical contact. The samples were then loaded into a Kratos Axis Ultra DLD spectrometer which possesses a base pressure below 1 x 10^{-10} mbar. XPS measurements were performed in the main analysis chamber at room temperature and at a take-off angle of 90° with respect to the surface parallel. The work function and binding energy scale of the spectrometer were calibrated using the Fermi edge and $3d_{5/2}$ peak recorded from a polycrystalline Ag sample prior to the commencement of the experiments. The samples were illuminated by a monochromatic Al Kα x-ray source (hν = 1486.7 eV) and with a low energy electron beam from a charge neutraliser in order to mitigate the build-up of positive charge on the surface during data collection. The core level spectra were recorded using a pass energy of 20 eV (resolution approx. 0.4 eV), from an analysis area of 300 x 700 μm. The data were analyzed in the Casa XPS package using Shirley backgrounds and mixed Gaussian-Lorentzian (Voigt) line shapes. During analysis, the binding energy scale was adjusted so that the C-C/C-H component in the C 1*s* region was located at a binding energy of 285.0 eV. For compositional analysis, the analyzer transmission function has been determined using clean metallic foils to determine the detection efficiency across the full binding energy range.

Raman spectra at room temperature were collected through a Renishaw inVia Reflex Raman Microscope equipped with DPSS laser at 633 nm (10% power nominally 2 mW) and Renishaw CCD detector. Objective of X50 LWD and an acquisition time of 10 s was used during testing.

Surface area measurements were performed using nitrogen adsorption via the Brunauer–Emmett–Teller (BET) method using a QUADRASORB (gas sorption surface area analyzer) (Quantachrome UK, Hook, United Kingdom) after degassing samples under vacuum at 150 °C for 12 hours.

NO_3RR measurements

The electrochemistry experiments for NO_3RR were conducted in a gas-tight H-type cell, in which the cathodic and anodic compartments were separated by a proton-exchange membrane (Nafion 211 membrane). Before use, the Nafion membrane was pretreated by boiling in a 5 % H_2O_2 aqueous solution at 80 °C for 1 h and subjected to multiple rinses. The counter-electrode is a Nickel foam and the reference electrode is a leak-free Ag/AgCl electrode (potential 0.198 V versus standard hydrogen electrode). The anodic electrolyte is 1 M KOH electrolyte (50 ml), while the cathodic electrolyte is 50 ml 1 M KOH + KNO_3 (1 M or 0.1 M). The electrolyte solutions were deaerated before the experiments by continuously bubbling argon with a 10 ml min^{-1} flow rate for 10 minutes to remove the N_2 and O_2. The reference electrode was immersed in a 3.5 M potassium chloride standard solution to calibrate its potential prior to the electrochemical measurements, by comparing the measured potential with the theoretical potential at room temperature. The LSV, chronoamperometric and chronopotentiometric measurements were conducted using a Solartron 1287. An electrochemical interface controlled by electrochemical software Corr-Ware/CorrView. Current densities were normalized to the

electrode's geometric surface area (1 cm^2). All potentials were calibrated to the RHE by the equation:

$$E_{RHE} = E_{\frac{Ag}{AgCl}} + E + 0.059 \times pH \quad (1)$$

Where $E_{Ag/AgCl}$ is 0.198 V vs RHE, E is the applied potential, the pH is 14 (1 M KOH).

Durability tests were conducted in an H-type cell using a three-electrode configuration (Ni foam and Ag/AgCl electrode as the counter electrode and reference electrode, respectively; Nafion 211 as the membrane) in 1 M KOH + 0.1 M KNO_3 electrolyte at current density of 200 mA cm^{-2} and 500 mA cm^{-2}, respectively.

ECSA analysis

We used the double-layer capacitance (C_{dl}) method to calculate the electrochemical active surface area (ECSA). The C_{dl} was obtained by measuring the capacitive current from cyclic voltammetry in a non-faradaic potential range and divided by the scan rate. The ECSA of the working electrodes was calculated according to the following equation:

$$I_c = vC_{dl} \quad (2)$$

$$ECSA = \frac{C_{dl}}{C_s} \quad (3)$$

where I_c represents the charging current at different scan rates, ν is the double-layer capacitance, and Cs is the specific capacitance in the range 20–60 μF cm^{-2} for a flat metallic surface. Here, we assume this is 40 μF cm^{-2}.

Preparation of electrode

catalyst ink was prepared by homogenizing 5mg catalyst and 5 mg conductive black carbon in 0.75mL isopropanol, 0.24mL H_2O, and 10 μL Nafion solution (5 wt%) through 1 h ultrasonication. The fully dispersed ink was dropped on a 1x1 cm^{-2} carbon cloth put on a 60 °C hotplate. The prepared work electrode with a loading mass of 2 mg cm^{-2} was used to investigate the surface-H effect and the stability test in 1 M KOH + 0.1 M KNO_3 electrolyte. The work electrode with a loading mass of 5 mg cm^{-2} was used to systematically investigate the electrocatalytic NO_3RR performances in 3 M KOH + 0.5 M KNO_3 electrolyte and Zn-NO_3 battery test. Chronoamperometric test was carried out to analyse the nitrogenous species (NO_3^-, NO_2^-, NH_3) Faradic efficiency and yield rate under different applied potentials.

The detection and quantification of Nitrogenous species (NO_3^-, NO_2^-, NH_3)

The Ammonia generated after chronoamperometry measurements with different potentials were detected and quantified through the sodium salicylate method. A certain amount of electrolyte was collected and diluted to the detection range. Then, 0.5 ml sodium salicylate solution consisting of 50 g L^{-1} sodium salicylate, 50 g L^{-1} potassium sodium tartrate and 20 g L^{-1} NaOH, 0.05 ml of 10 g L^{-1} sodium nitroprusside solution and 0.05 ml sodium hypophosphite solution contained 40 ml L^{-1}, 13 wt% NaClO and 30 g L^{-1} NaOH were added to 5 ml diluted electrolyte in order. After 20 minutes, UV-vis spectrophotometer (Shimadzu UV-2600) was used to collect the adsorption spectrum and quantify the concentration of the generated NH_3 in the electrolyte based on the calibrated curve.

The byproduct nitrite was identified and quantified using UV-vis spectrophotometry. A certain amount of electrolyte was collected and diluted to the detection range. Subsequent, 0.1 ml of

nitrite colour reagent consisted of 40 g L^{-1} p-aminobenzene sulfonamide, 100 ml L^{-1} phosphoric acid and 2 g L^{-1} N(1-naphthyl)-ethylenediamine dihydrochloride was added to the 5 ml diluted solution. After 20 min, the absorption spectrum was collected using a UV-vis spectrophotometer, and the absorption intensities at a wavelength of 500 nm were recorded. The concentration–absorbance curve was calibrated using a series of standard potassium nitrite solution, and the potassium nitrite crystal was dried at ~105-110 °C for 2 h in advance.

The nitrogenous species yield rate (Y) and Faradic efficiency (FE) were calculated according to the following equation:

$$Y_{NH_3} = \frac{c \times V}{S \times t} \tag{4}$$

$$FE_{NH_3} = \frac{n \times V \times F}{Q} \tag{5}$$

where n is the electron-transfer number (8 for 1mol NH_3, 2 for 1mol NO_2^-), V is the volume of the catholyte of the catholyte of the cathode chamber (50mL), c represents the concentration of the outlet products (M), S is the area of the electrode, F is the Faraday constant (96,485 C/mol), and Q represents the applied overall coulomb quantity (C).

The Energy efficiency (EE) of half-reaction was calculated according to the following equation:

$$EE = \frac{(E^{\theta}_{OER} - E^{\theta}_{NH3}) \times FE_{NH3}}{E^{\theta}_{OER} - E_{NH3}} \tag{6}$$

where E^{θ}_{NH3} represents the equilibrium potential of nitrate electroreduction to NH_3 (0.69 V versus RHE under alkaline conditions), E^{θ}_{OER} is the equilibrium potential of the oxygen evolution reaction (OER) (1.23 V versus RHE), FE_{NH3} is the Faradaic efficiency for NH_3, and E_{NH3} is the applied potential.

The detection and quantification of H_2

In this work, the Faradaic efficiency for H_2 (FE_{H2}) was indirectly estimated based on the total charge passed and the quantified liquid-phase products (NH_3 and NO_2^-). Specifically, the fraction of charge not accounted for by these detected products was attributed to H_2 evolution. This mass-balance-based approach is commonly adopted in

This method may introduce some uncertainty, as it assumes that H_2 is the major undetected product and does not explicitly exclude the presence of other minor species. Therefore, the reported FE_{H2} values should be considered as semi-quantitative estimations rather than exact measurements.

However, in this work, the FE_{H2} values are mainly used for comparative analysis across different catalysts under identical conditions, rather than for absolute quantification. Therefore, the relative trends—particularly the suppressed HER on $FeFeCoO_4$—remain reliable and meaningful.

DFT computational details

All calculations were performed based on density functional theory, with the Perdew–Burke−Ernzerhof (PBE) exchange-correlation functional, and the projected augmented wave (PAW) scheme was implemented in a Vienna Ab initio Simulation Package (VASP) code[1]. The cut-off energy of the plane wave function was set to 500 eV. The Brillouin zone was sampled with a 6 × 6 × 6 and 4 × 3 × 1 Monkhorst–Pack mesh for bulk models of $Co_XFe_{3-x}O_4$ (x=0,0.5, 1) and $Co_xFe_{3-x}O_4$ (110), respectively. The convergence criterion for the energy and the force tolerance was set to 10-5 eV and -0.02 eV Å−1 on each atom, respectively. DFT+U were considered, and the effective Hubbard parameter (U-J) for the Fe 3d states and Co 3d is taken as 3.8 eV as was employed in previous studies[2].

The simulation models were constructed based on experimental characterizations. To investigate the NO_3RR activities of $FeFeCoO_4$, FeFeCoO (110) facet with 4 atom layers was built, of which the bottom two layers were fixed and the uppermost layers as well as the adsorbed species were allowed to relax. The boundary conditions were set to be periodic in both the x and y directions, while a 15 Å vacuum separated the surfaces along the z direction.

The Gibbs free energy change (ΔG) of each element step was calculated using the computational hydrogen electrode (CHE) model[3], which uses one-half of the chemical potential of hydrogen as the chemical potential of the proton-electron pair. Then, the ΔG value can be determined as follows:

$$\Delta G = \Delta E + \Delta ZPE - T\Delta S + \Delta G_U \qquad (7)$$

where ΔE is the electronic energy difference of catalyst directly obtained from DFT calculations, ΔZPE is the change in zero-point energies, T is the temperature (T = 298.15 K), and ΔS is the entropy change. ΔG_U is the free energy contribution related to electrode potential U. The zero-point energies and entropies of the adsorption species were computed from the vibrational frequencies, in which only the vibrational modes of adsorbate were explicitly calculated, while the catalyst sheet remained fixed. The entropies and vibrational frequencies of molecules in the gas phase were taken from the NIST database.

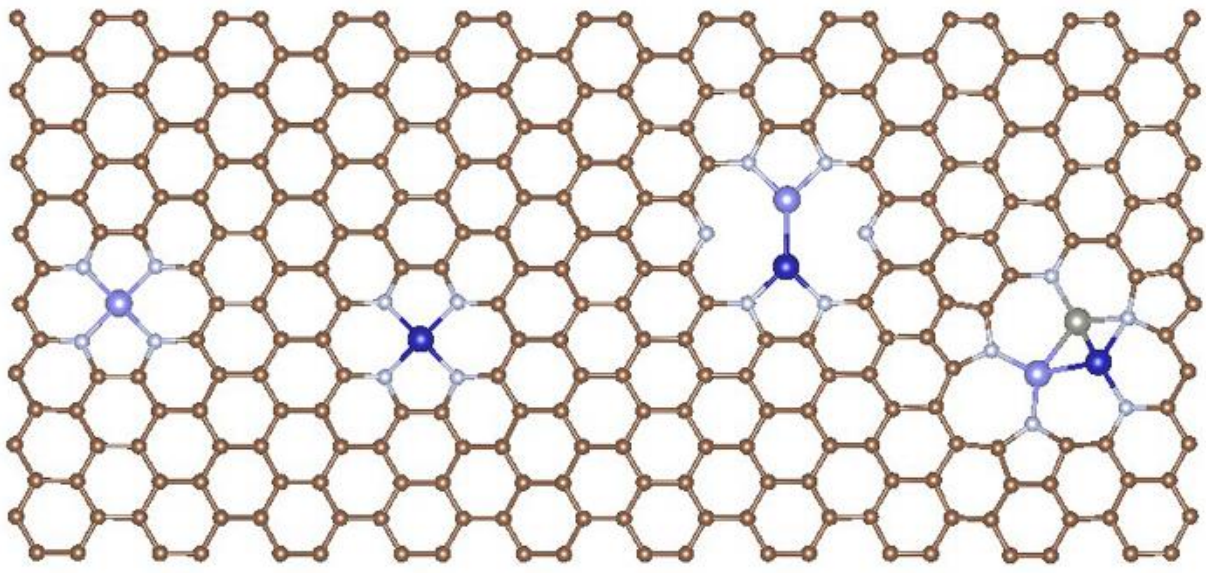

Figure S1. Schematic of multi-metal atom catalysts. The coordination environment of metal atoms is variable, uncertain and uncontrolled. It is difficult to construct abundant ICM consisted of both *H generation site and decoupled adsorption-activation centers in these catalysts,

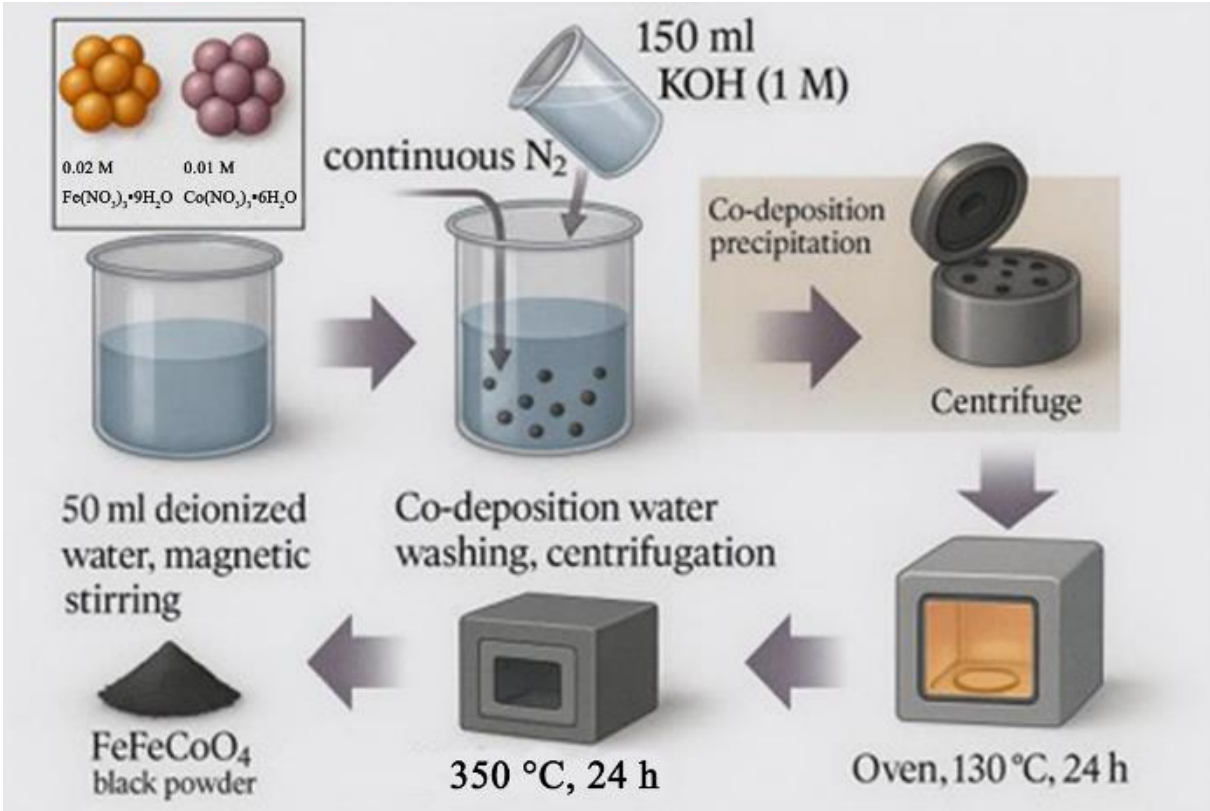

Figure S2. Schematic of preparation method of $FeFeCoO_4$ spinel oxide.

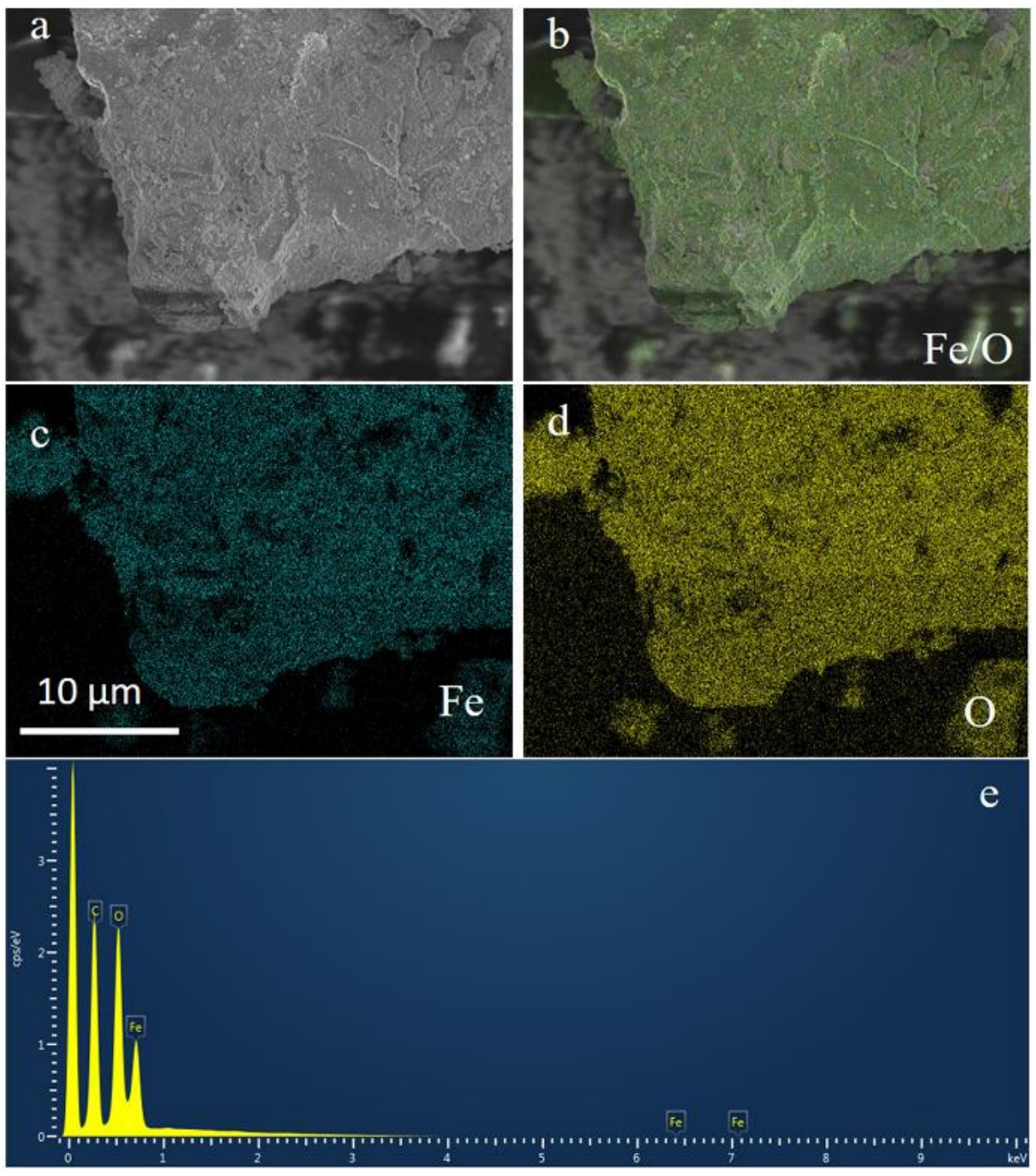

Figure S3. Structure characterization of Fe_3O_4. SEM image (a) of Fe_3O_4 and corresponding elements mapping analysis, (b) total (c) Fe, and (d) O. **e,** the corresponding EDS spectrum.

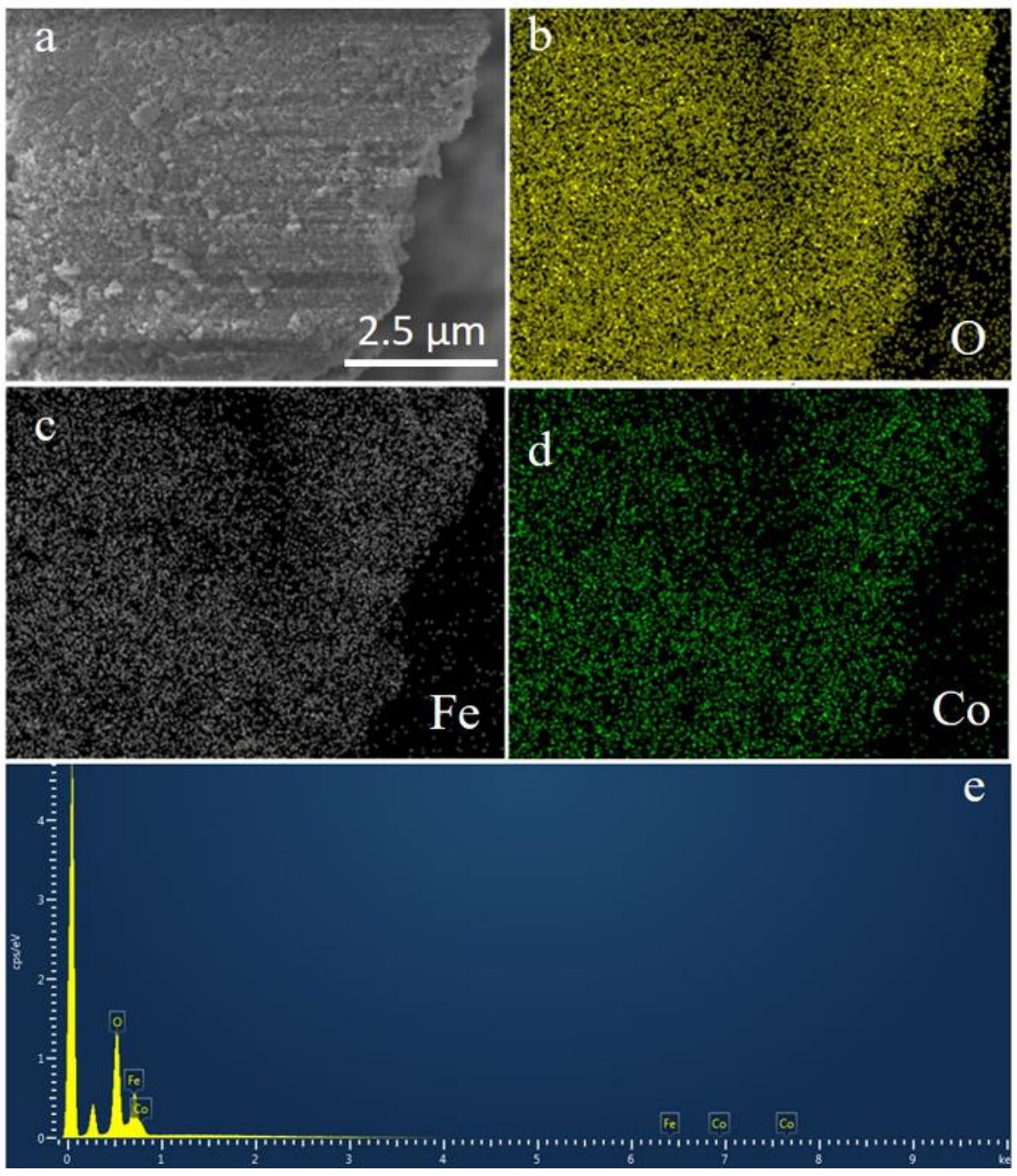

Figure S4. Structure characterization of $FeFe_{1.5}Co_{0.5}O_4$. SEM image (a) of $FeFe_{1.5}Co_{0.5}O_4$ and corresponding elements mapping analysis, (b) O (c) Fe, and (d) Co. **e,** the corresponding EDS spectrum.

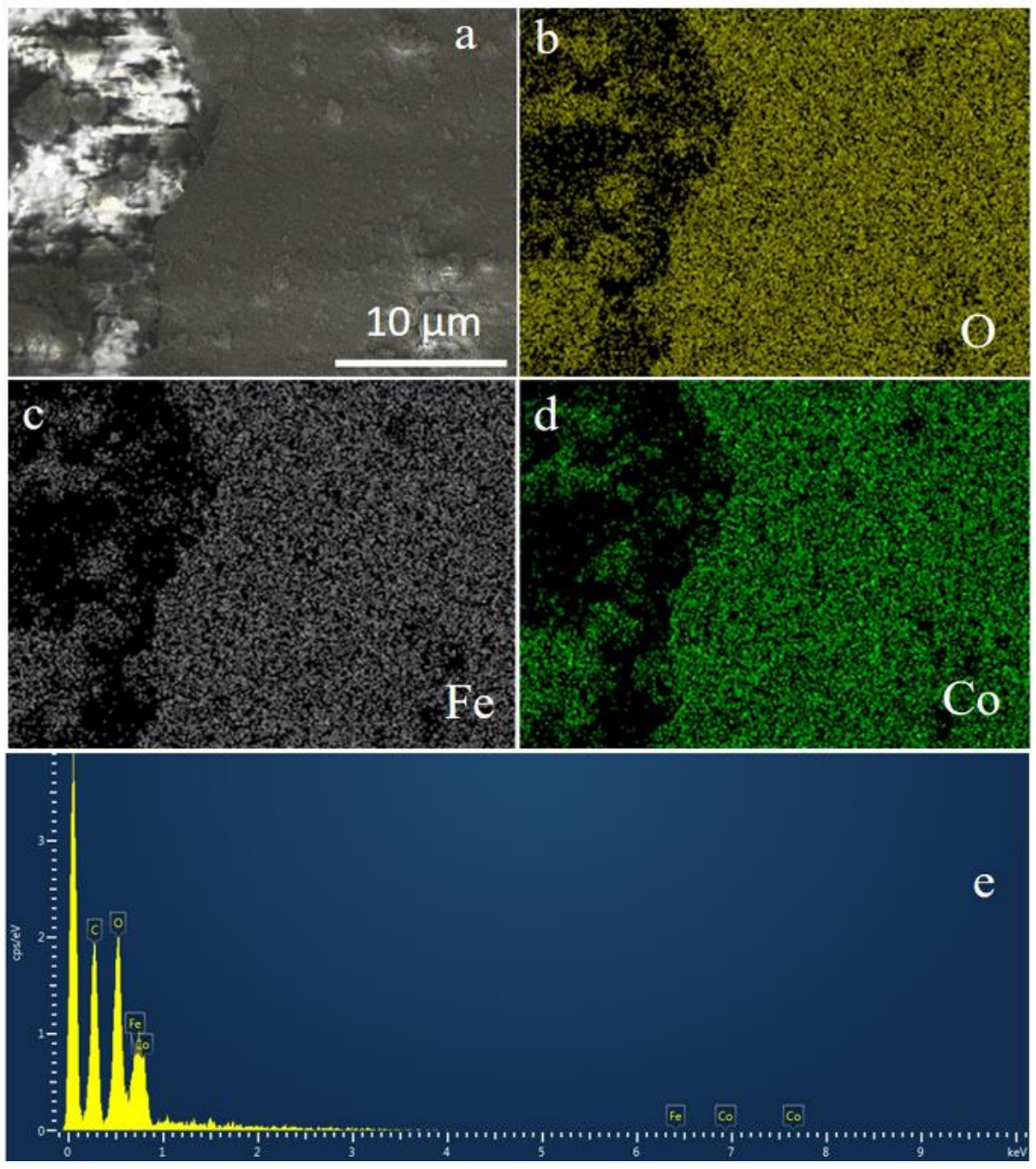

Figure S5. Structure characterization of $FeFeCoO_4$. SEM image (a) of $FeFeCoO_4$ and corresponding elements mapping analysis, (b) O (c) Fe, and (d) Co. **e,** the corresponding EDS spectrum.

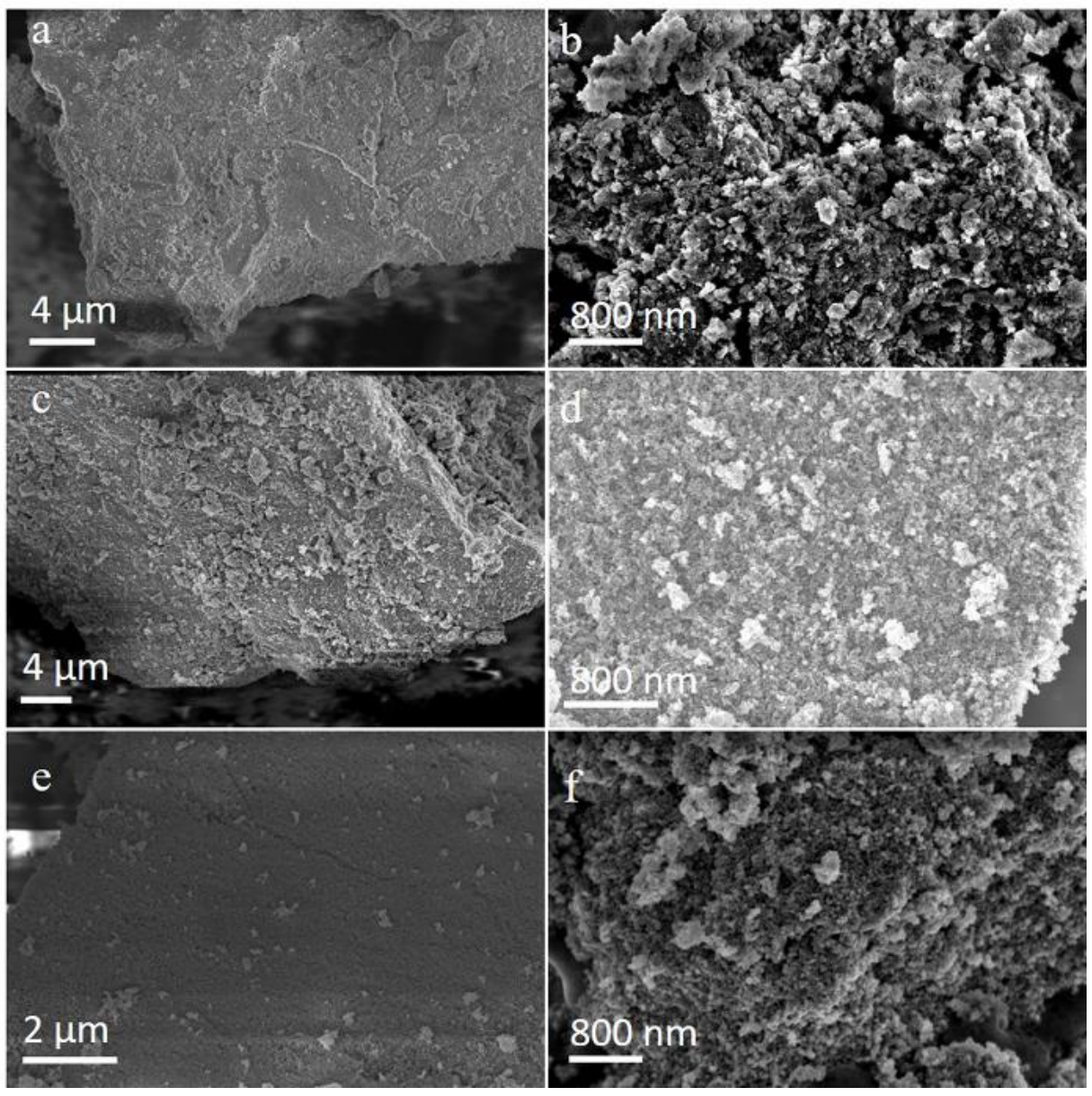

Figure S6. Morphology structure of $Co_xFe_{3-x}O_4$ spinel oxides. SEM images of Fe_3O_4 (a,b), $FeFe_{1.5}Co_{0.5}O_4$ (c,d) and $FeFeCoO_4$ (e,f).

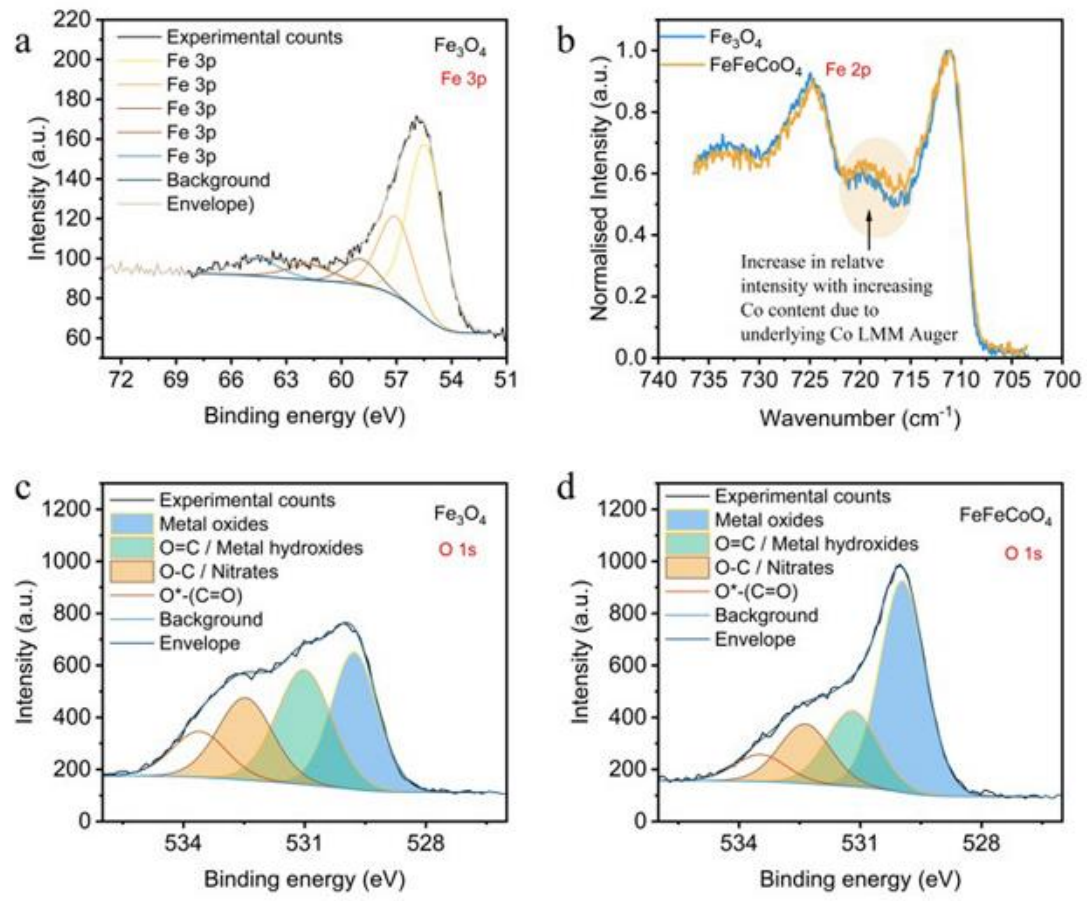

Figure S7. XPS patterns for Fe_3O_4 and $FeFeCoO_4$. a, Fe 3p for Fe_3O_4. **b,** full spectra of Fe 2p for $FeFeCoO_4$ and Fe_3O_4. O 1s XPS patterns for $FeFeCoO_4$ (**c**) and Fe_3O_4 (**d**).

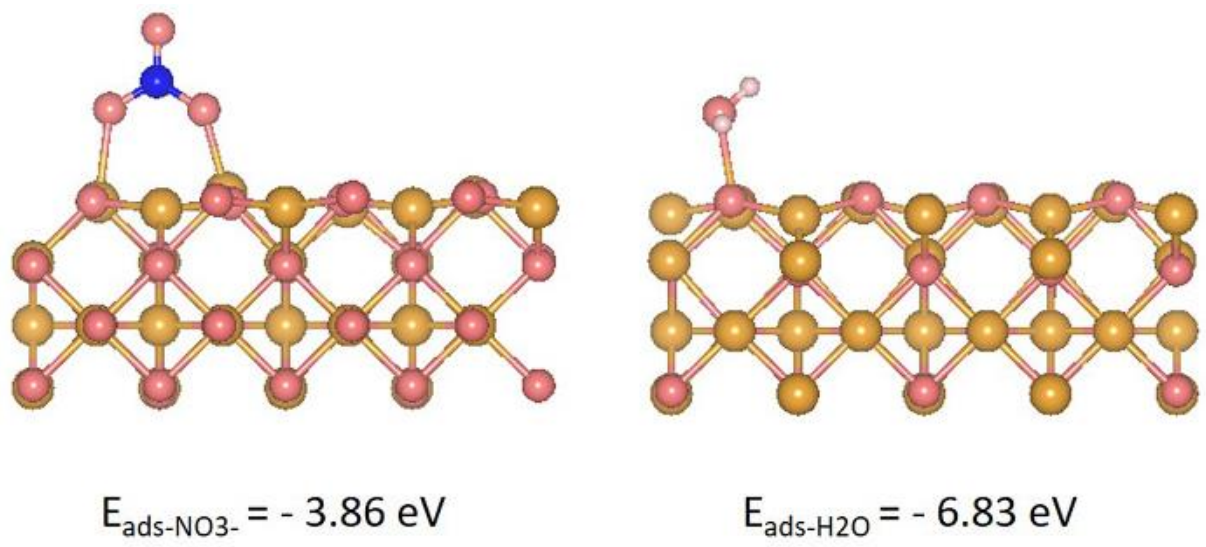

Figure S8. Adsorption energy analysis of NO_3^- and H_2O at the octahedral site on Fe_3O_4 (110) spinel oxides. The adsorption energy of H_2O is significantly higher than that of NO_3^-, thereby hindering NO_3^- adsorption and subsequent reduction.

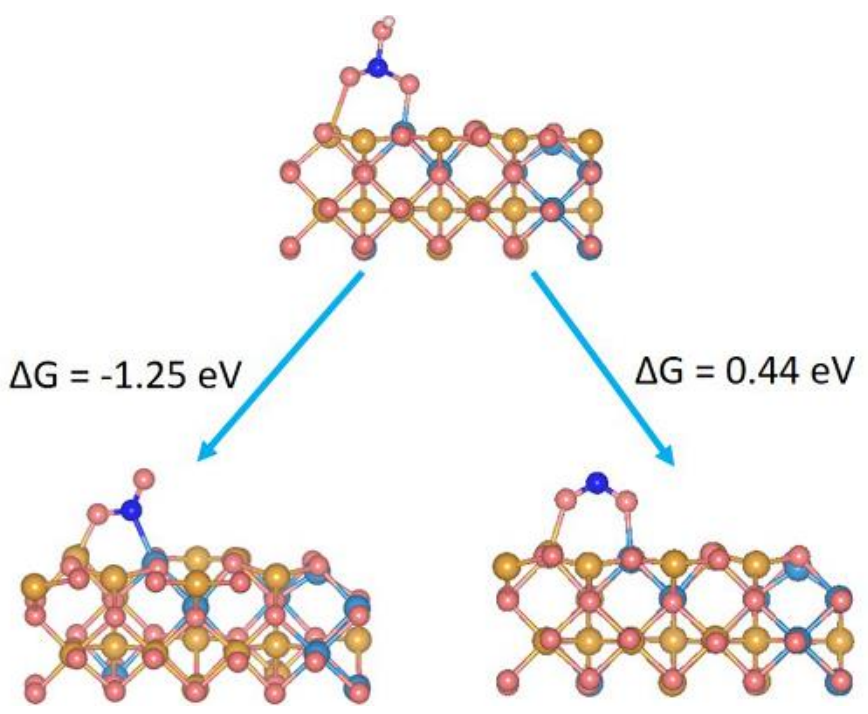

Figure S9. Adsorption configurations of $*NO_2$ on $FeFeCoO_4$ (110) spinel oxides. The Fe–O–N–Co configuration exhibits a lower Gibbs free energy than the Fe–O–N–O–Co configuration, highlighting the role of hard–soft acid dual-metal sites as decoupled activation–adsorption centers for NO_3RR.

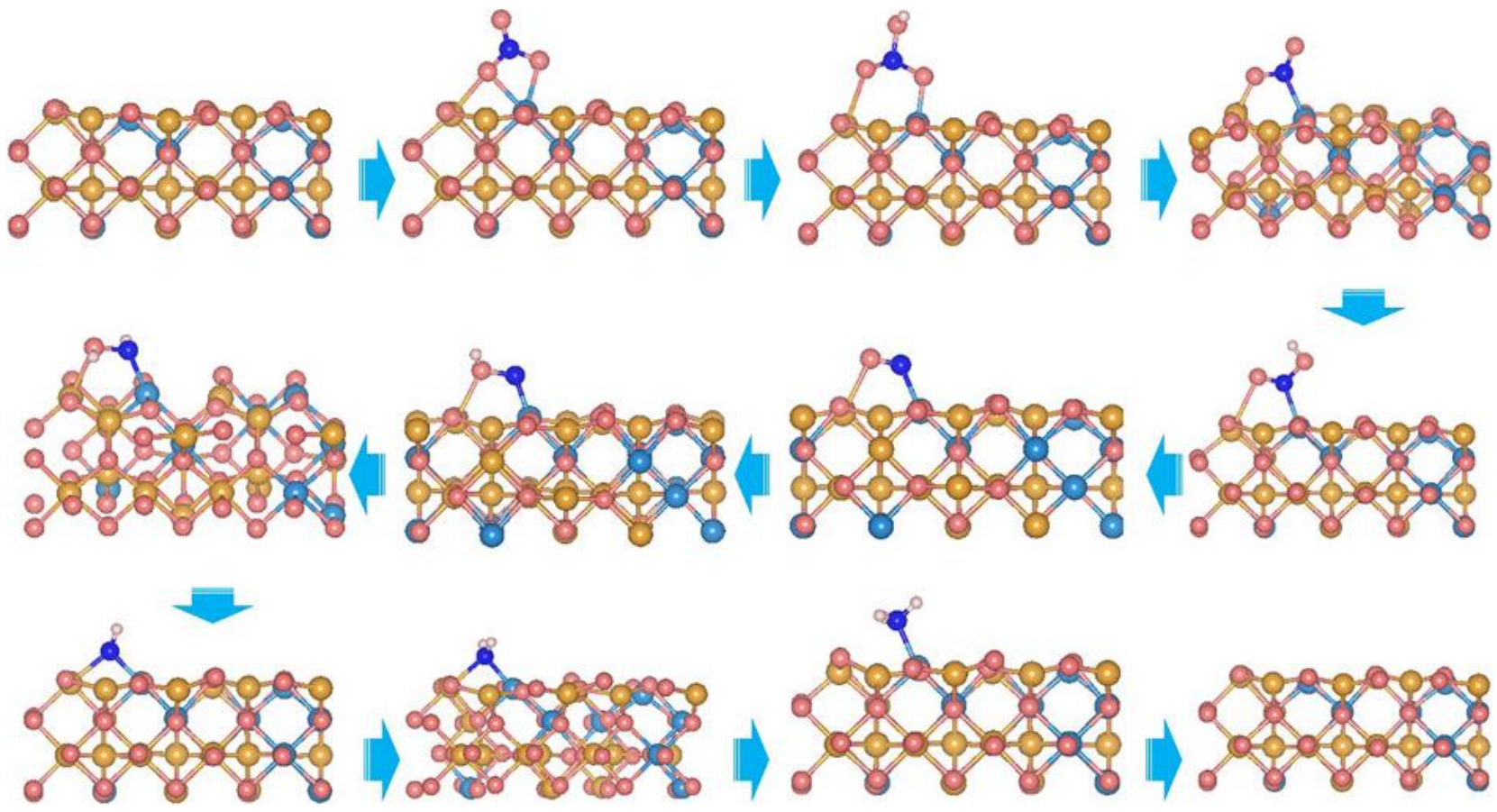

Figure S10. Reaction pathway of NO_3RR with corresponding intermediate configurations on $FeFeCoO_4$ (110) spinel oxides.

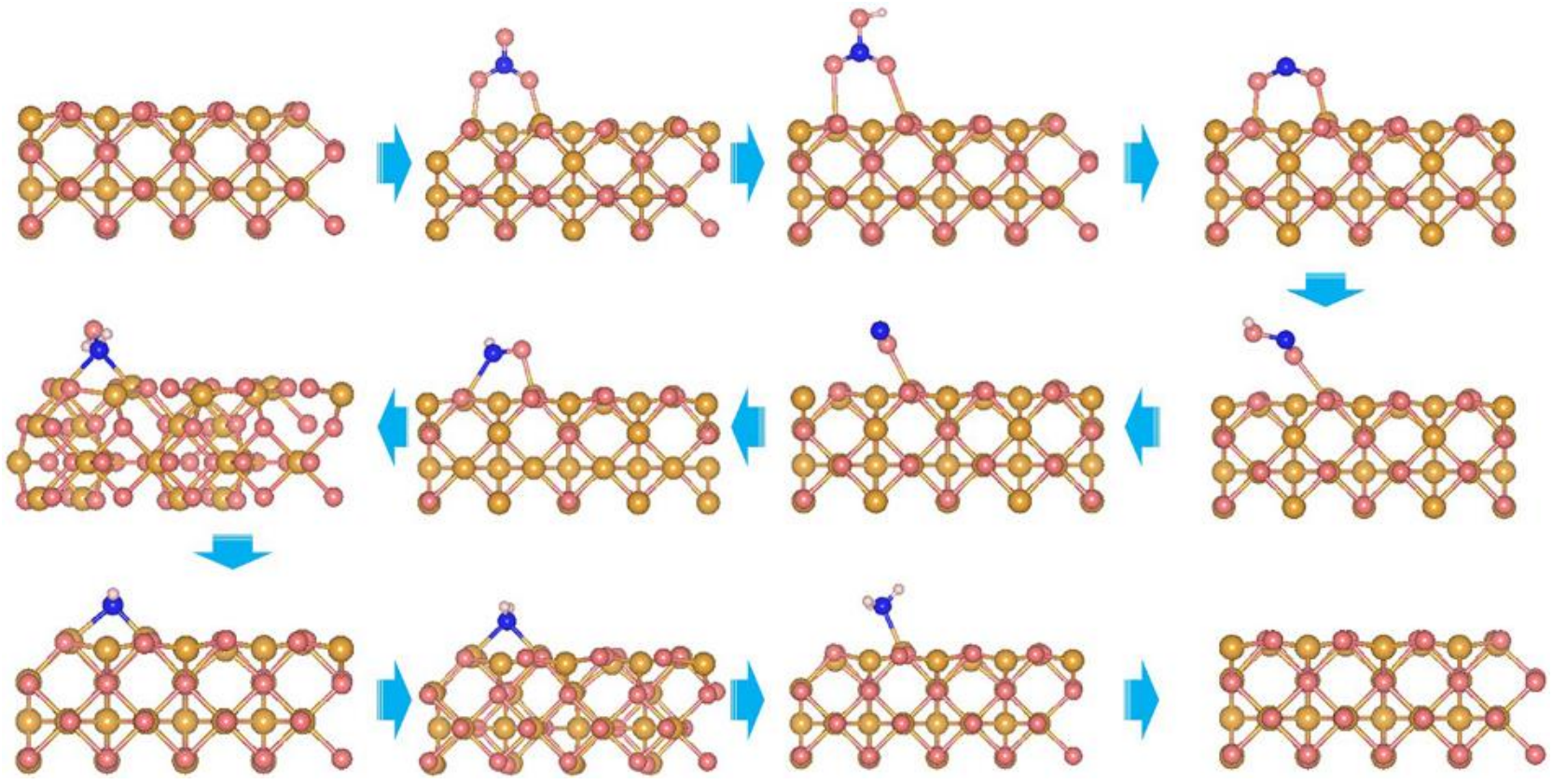

Figure S11. Reaction pathway of NO_3RR with corresponding intermediate configurations on Fe_3O_4 (110) spinel oxides.

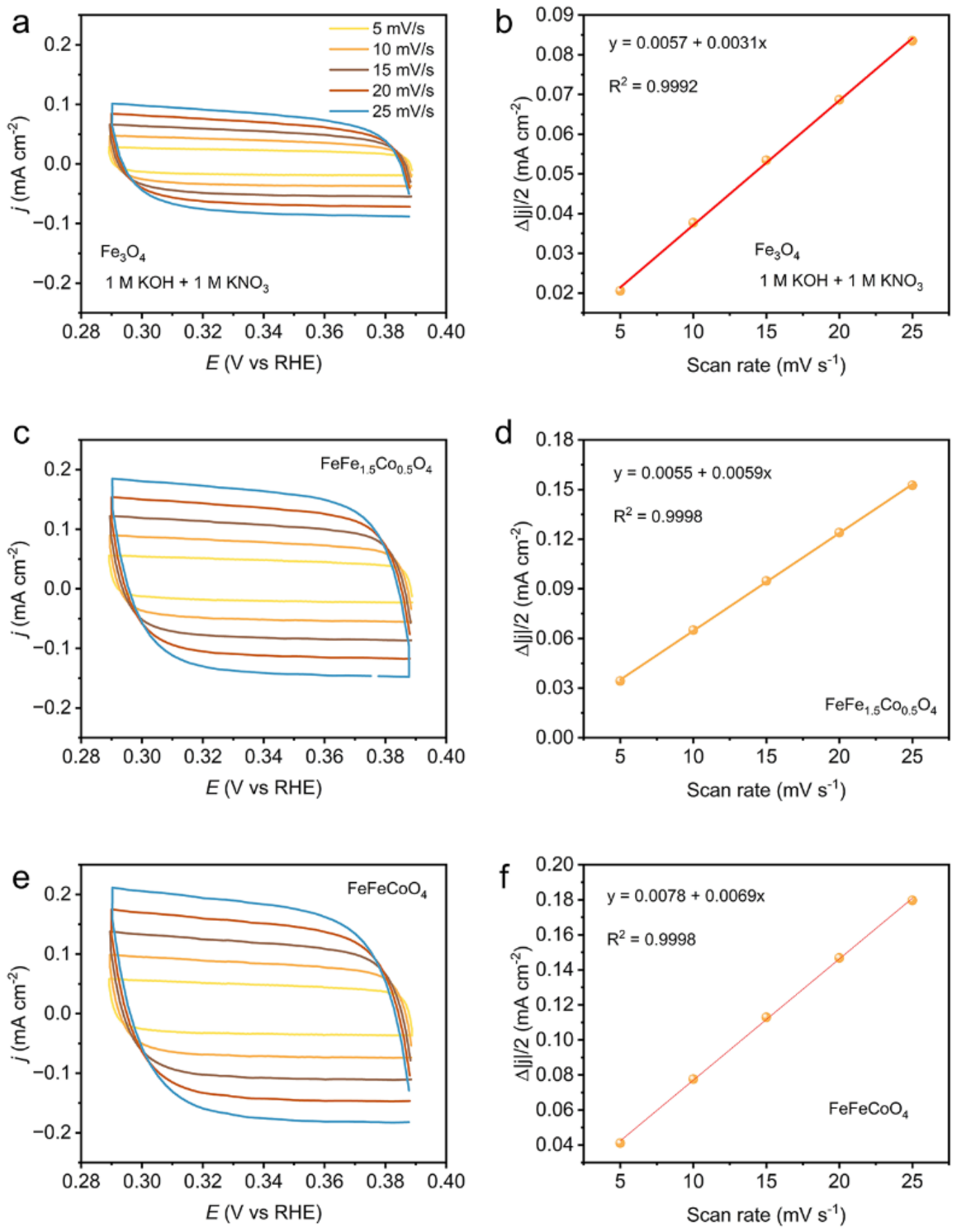

Figure S12. **ECSA analysis of $Co_xFe_{3-x}O_4$ spinel oxides.** CV curves of Fe_3O_4 (a), $FeFe_{1.5}Co_{0.5}O_4$ (c) and $FeFeCoO_4$ (e) with various scan rate from 5 to 25 mV s^{-1} and the corresponding C_{dl} calculation results of Fe_3O_4 (b), $FeFe_{1.5}Co_{0.5}O_4$ (d) and $FeFeCoO_4$ (f). The C_{dl} is calculated to be 3.1, 5.9 and 6.9 mF for Fe_3O_4, $FeFe_{1.5}Co_{0.5}O_4$ and $FeFeCoO_4$. Assuming the Cs of those catalysts as 40 μF cm^{-2}, the ECSA of Fe_3O_4, $FeFe_{1.5}Co_{0.5}O_4$ and $FeFeCoO_4$ are calculated as 77.5, 147.5, and 172.5 cm^{-2}, respectively.

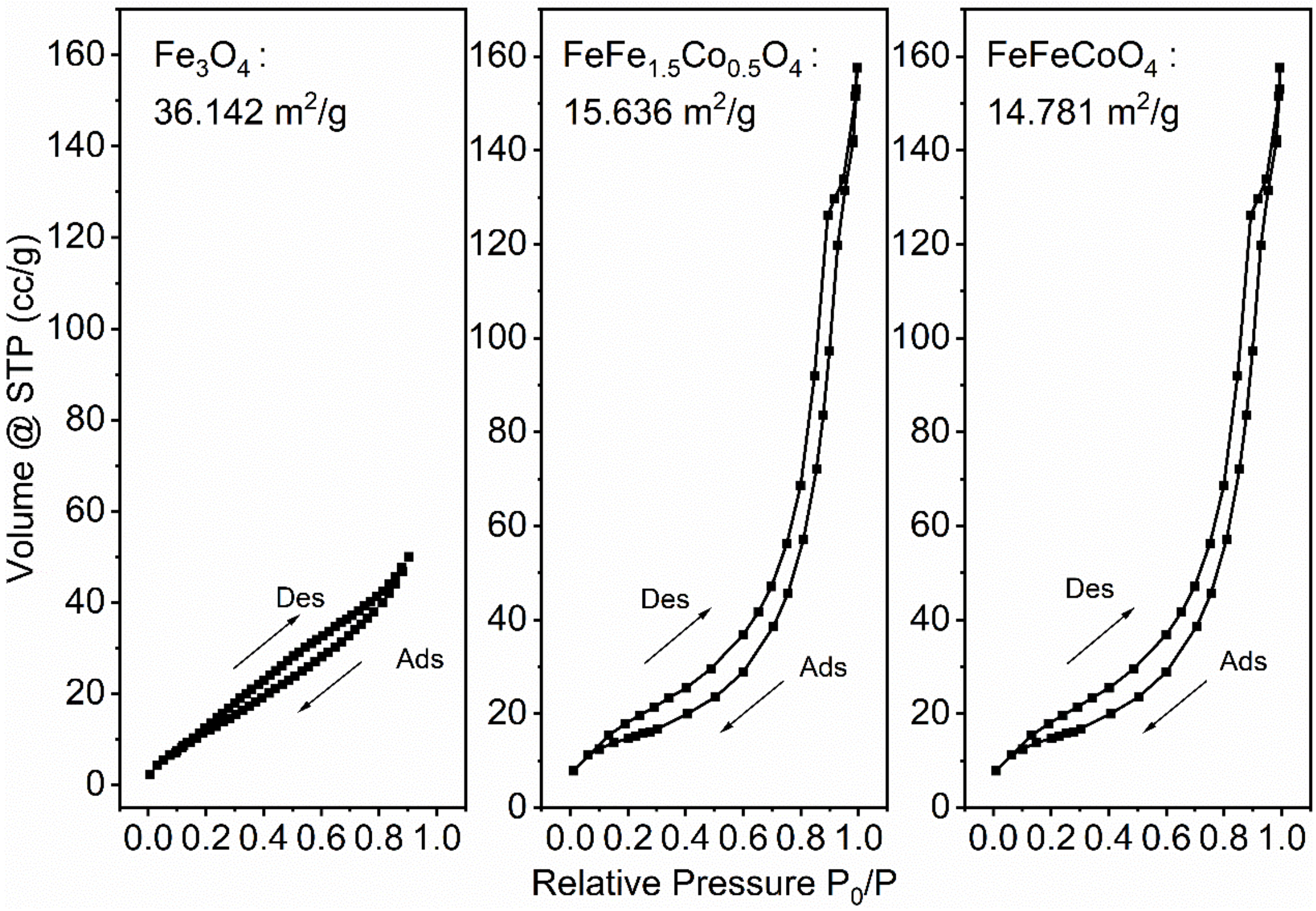

Figure S13. The BET lines and the corresponding BET values of the three samples (Fe_3O_4, $FeFe_{1.5}Co_{0.5}O_4$, and $FeFeCoO_4$) .

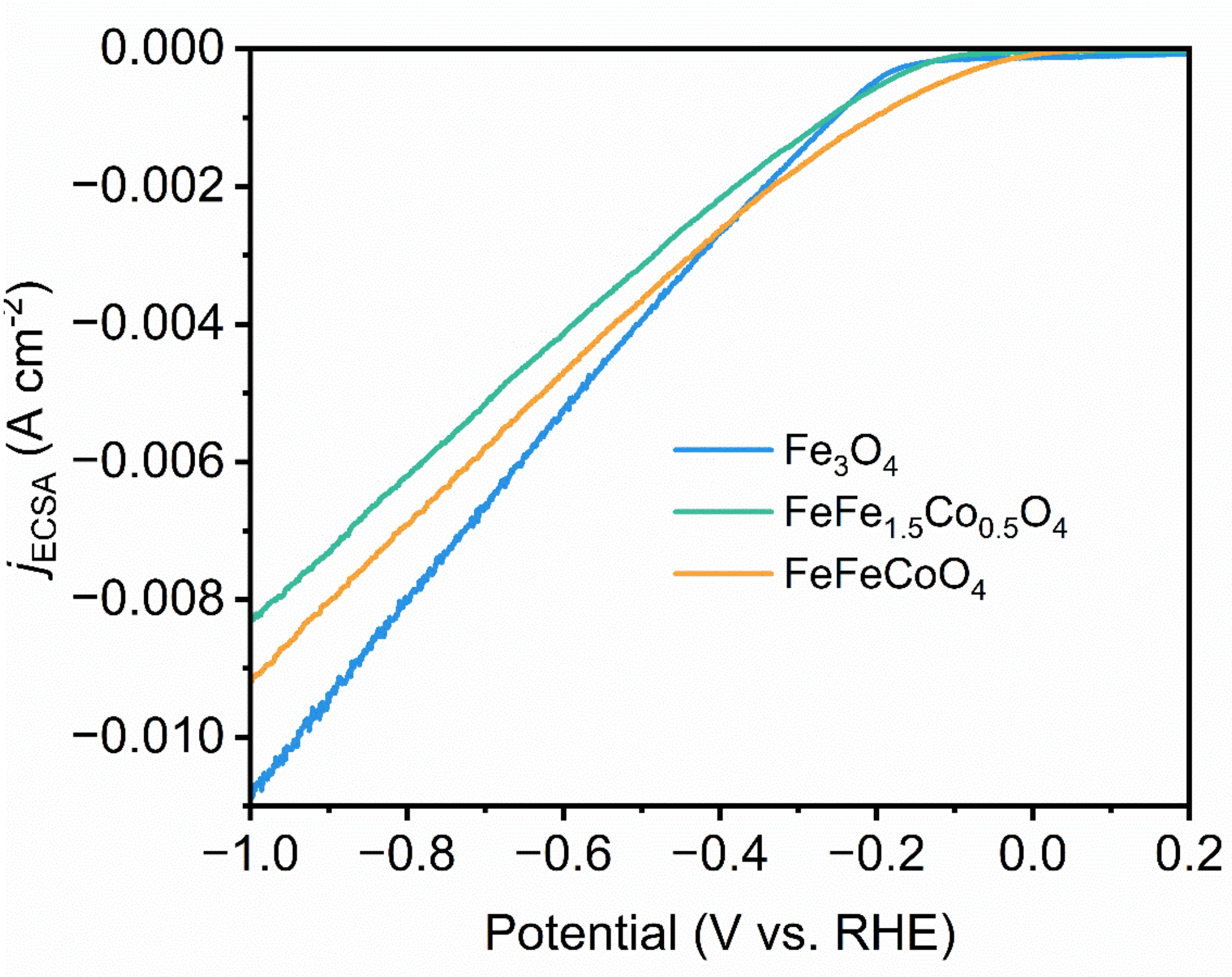

Figure S14. The ECSA-normalized current density of the three samples (Fe_3O_4, $FeFe_{1.5}Co_{0.5}O_4$, and $FeFeCoO_4$) under 1M KOH +1 M KNO3 electrolyte.

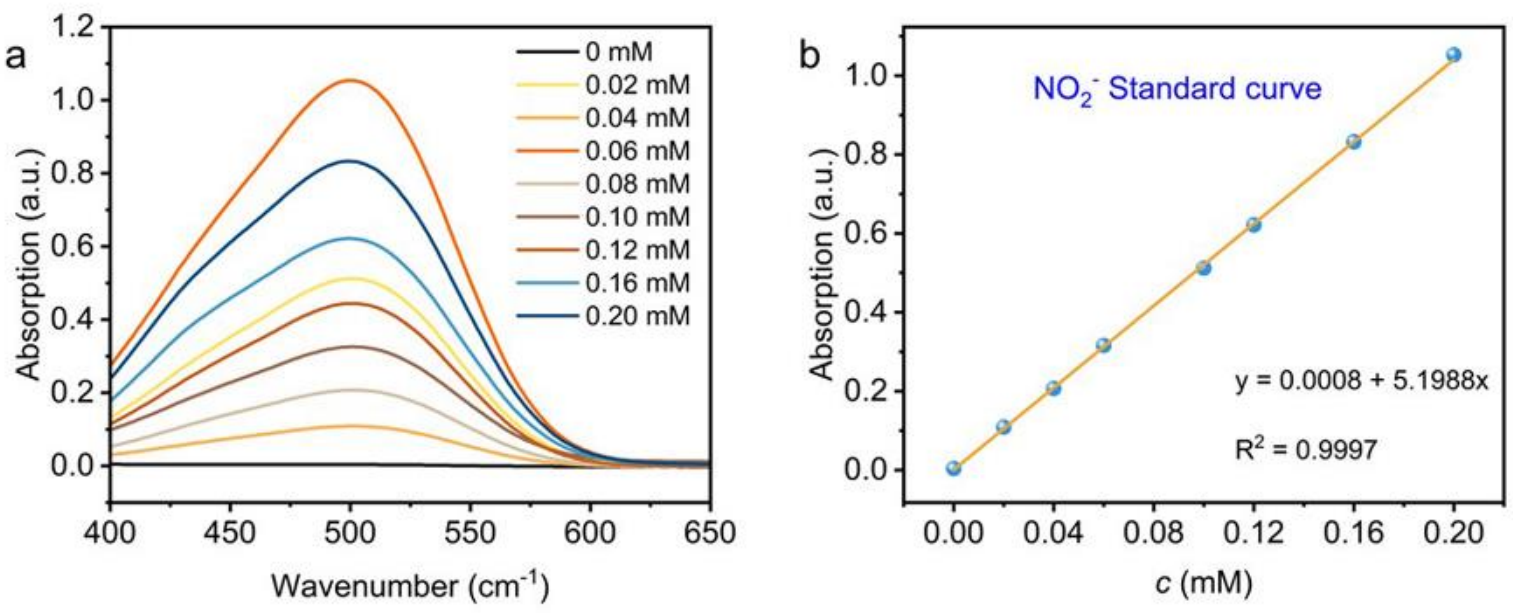

Figure S15. Standard curves of NO_2^- quantification by the colouration method. UV-vis absorption spectroscopy of diazotization spectrophotometry with different concentrations of nitrite-N (**a**) and the corresponding standard curve (**b**). Potassium nitrite was used as nitrite-N sources for standard curve preparation.

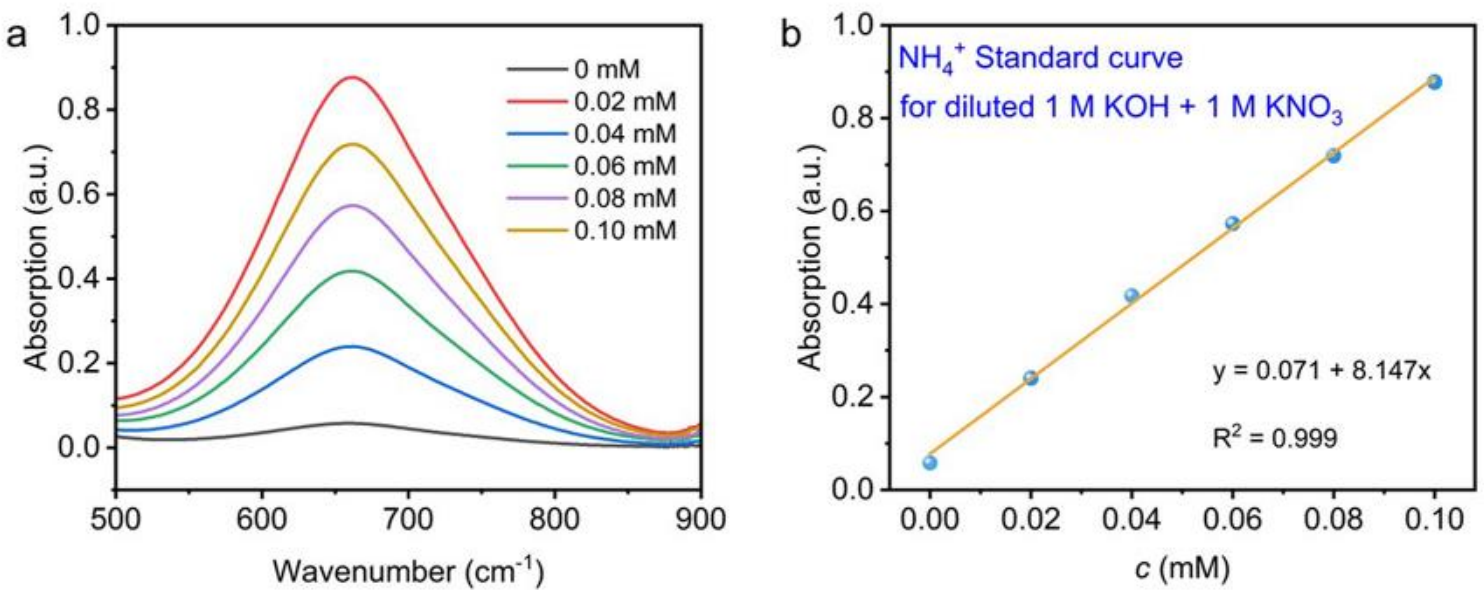

Figure S16. Standard curves of NH_4^+ quantification by the colouration method. UV-vis absorption spectroscopy of indophenol blue spectrophotometry with different concentrations of ammonia-N (**a**) and the corresponding standard curve (**b**). Ammonium chloride was used as ammonia-N sources for standard curve preparation. This standard curve used for the determination of ammonia in diluted electrolyte (1 M KOH + 1 M KNO_3) after chronoamperometry test under different potential for 0.5 h.

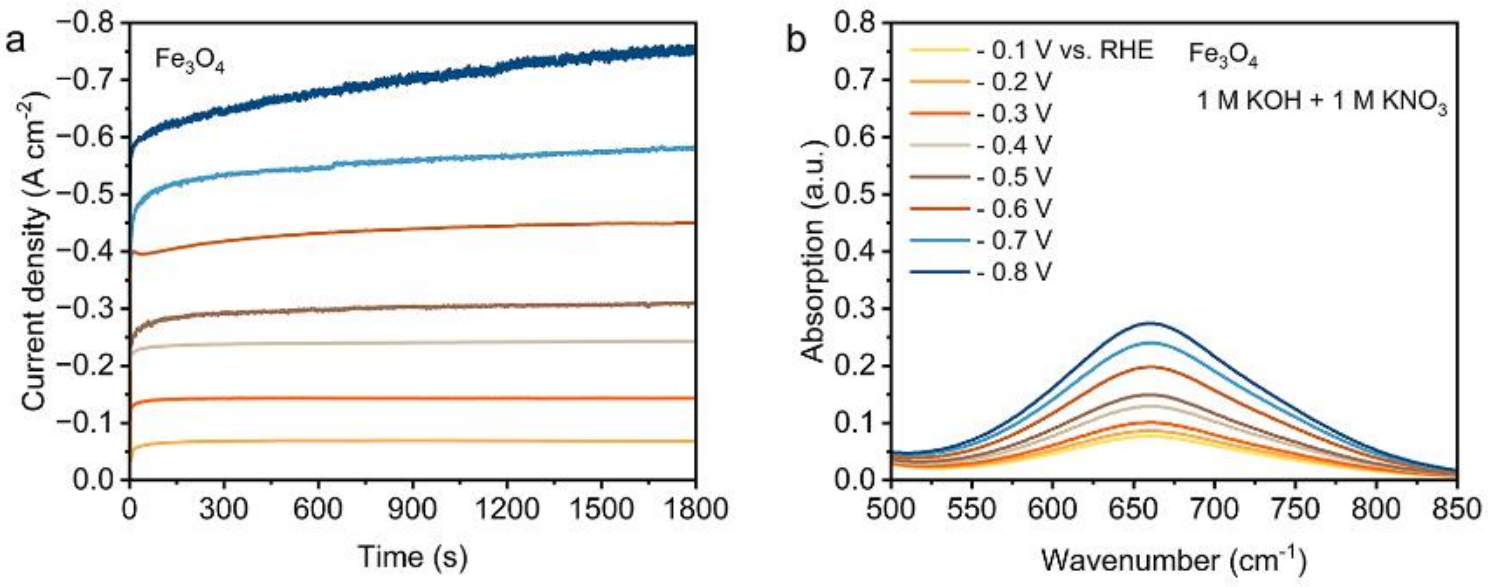

Figure S17. Quantification analysis of the generated ammonia after chronoamperometry measurements with different potentials over Fe_3O_4. The current density curves of chronoamperometry measurements at different potentials (vs. RHE) over Fe_3O_4 (a) and the corresponding UV-vis absorption spectroscopy of indophenol blue spectrophotometry.

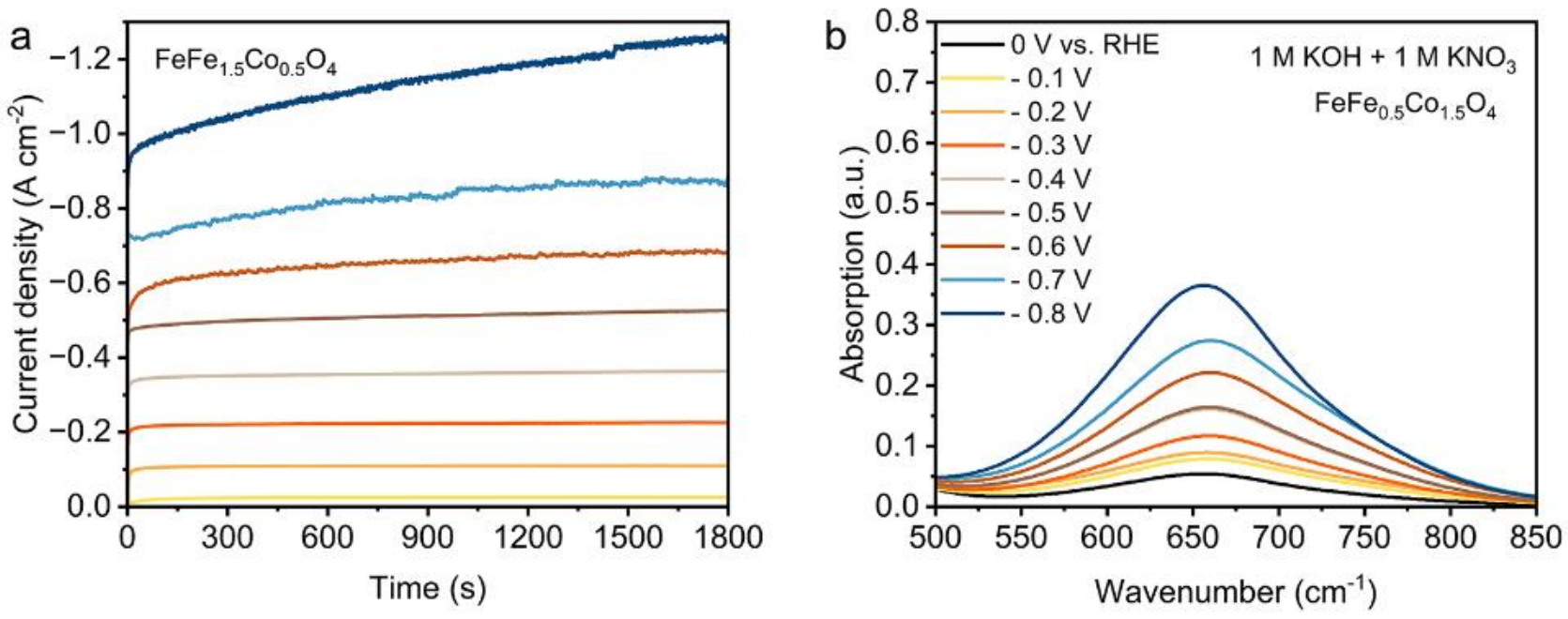

Figure S18. Quantification analysis of the generated ammonia after chronoamperometry measurements with different potentials over $FeFe_{1.5}Co_{0.5}O_4$. The current density curves of chronoamperometry measurements at different potentials (vs. RHE) over $FeFe_{1.5}Co_{0.5}O_4$ (a) and the corresponding UV-vis absorption spectroscopy of indophenol blue spectrophotometry.

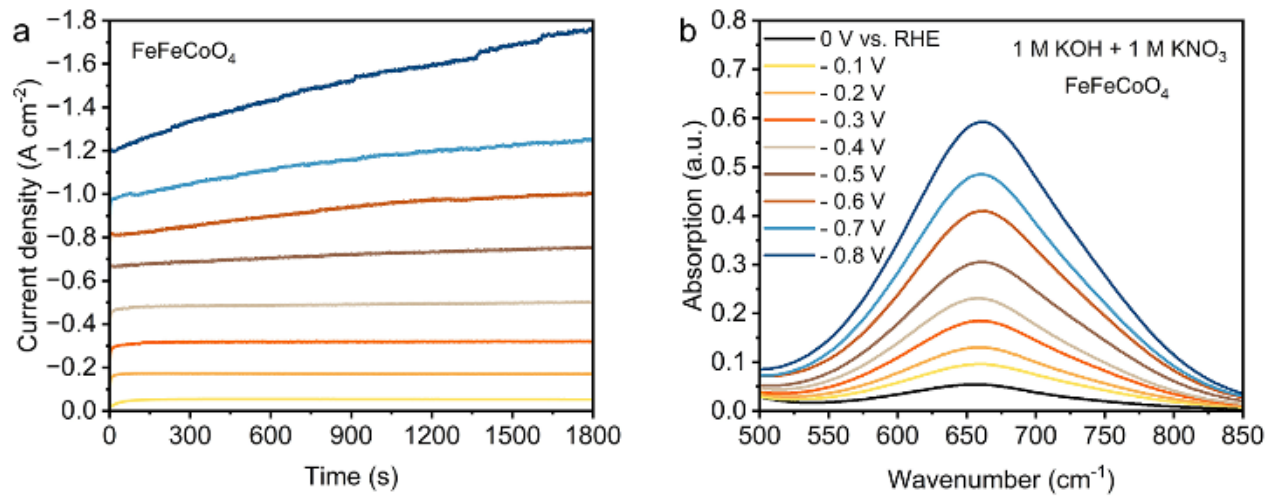

Figure S19. Quantification analysis of the generated ammonia after chronoamperometry measurements with different potentials over FeFeCoO4. The current density curves of chronoamperometry measurements at different potentials (vs. RHE) over FeFeCoO4 (a) and the corresponding UV-vis absorption spectroscopy of indophenol blue spectrophotometry.

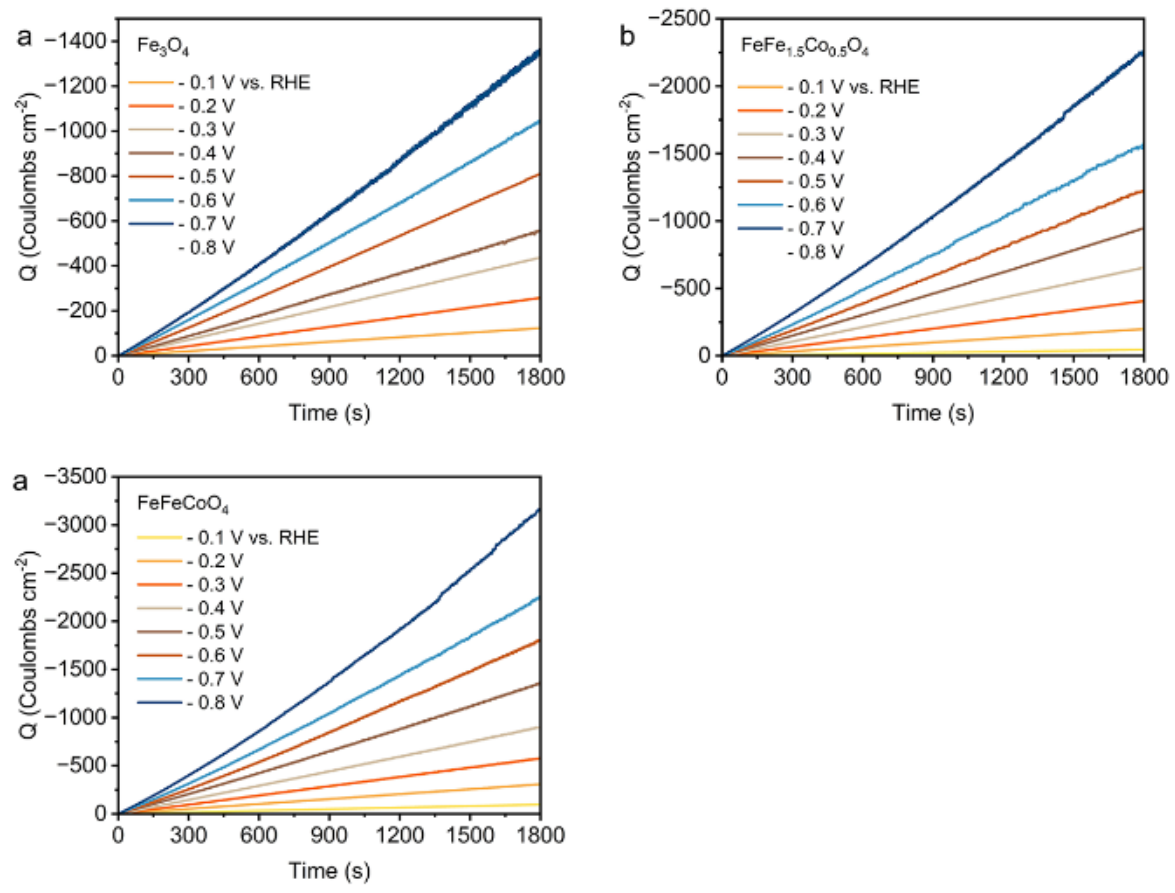

Figure S20. **Coulombs of the chronoamperometry measurements over $Co_xFe_{3-x}O_4$ spinel oxides.** (a) Fe_3O_4, (b) $FeFe_{1.5}Co_{0.5}O_4$ and (c) $FeFeCoO_4$. Coulomb is the key parameter for calculating the faradic efficiency.

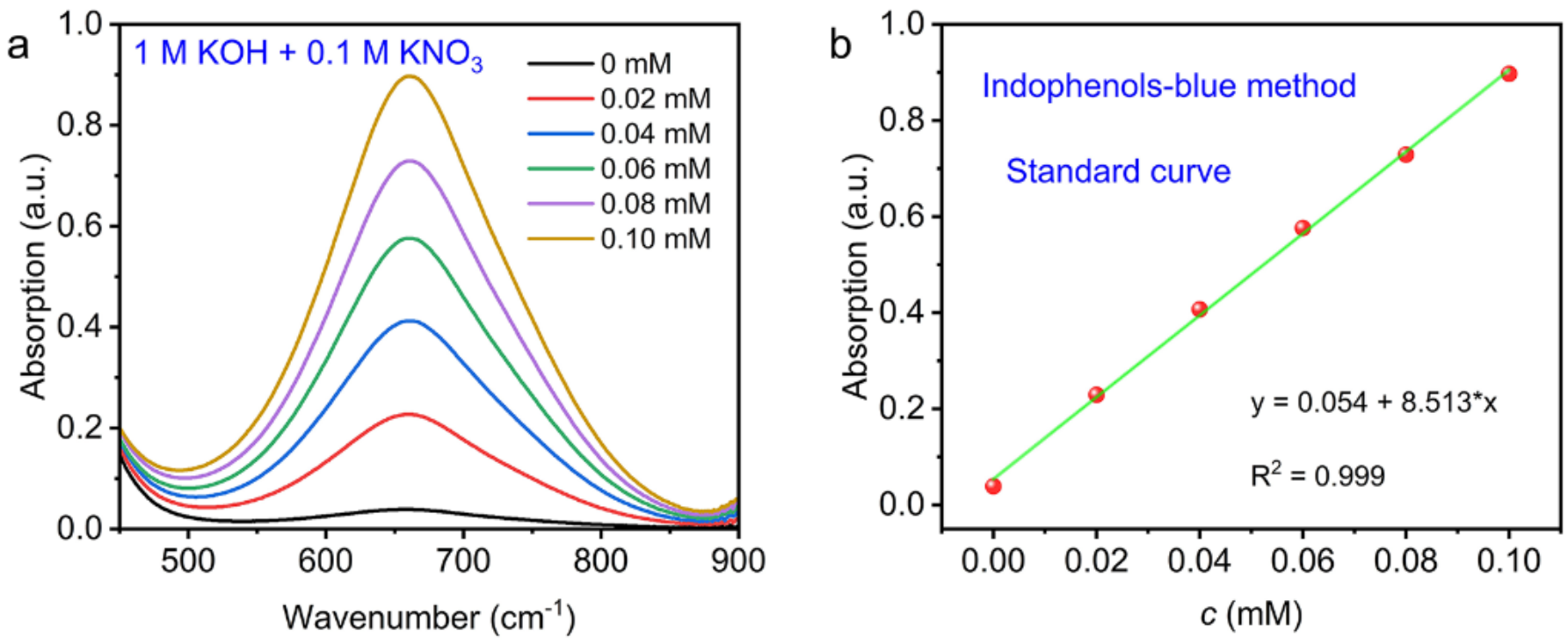

Figure S21. Standard curves of NH_4^+ quantification by the colouration method. UV-vis absorption spectroscopy of indophenol blue spectrophotometry with different concentrations of ammonia-N (**a**) and the corresponding standard curve (**b**). Ammonium chloride was used as ammonia-N sources for standard curve preparation. This standard curve used for the determination of ammonia in diluted electrolyte (1 M KOH + 0.1 M KNO_3) after chronoamperometry test under different potential for 1 h.

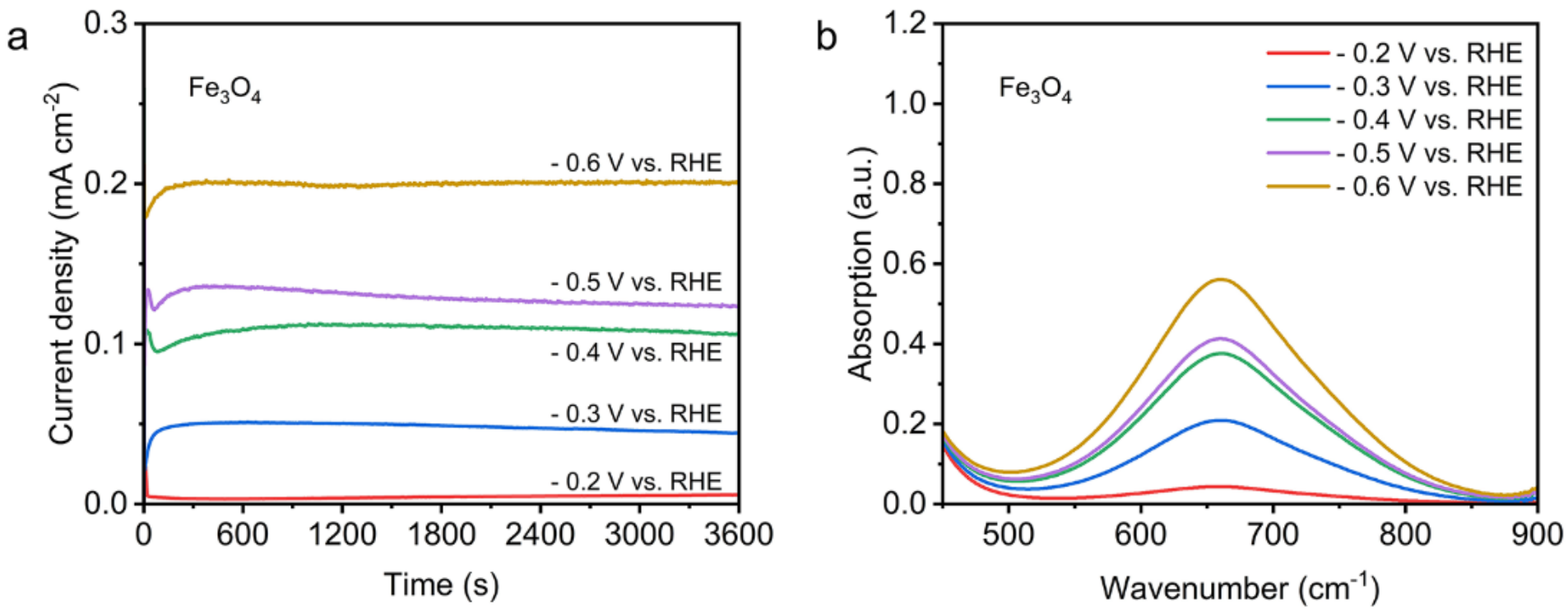

Figure S22. Quantification analysis of the generated ammonia after chronoamperometry measurements with different potentials in 1 M KOH + 0.1 M KNO_3 electrolyte over Fe_3O_4. The current density curves of chronoamperometry measurements at different potentials (vs. RHE) over Fe_3O_4 (a) and the corresponding UV-vis absorption spectroscopy of indophenol blue spectrophotometry.

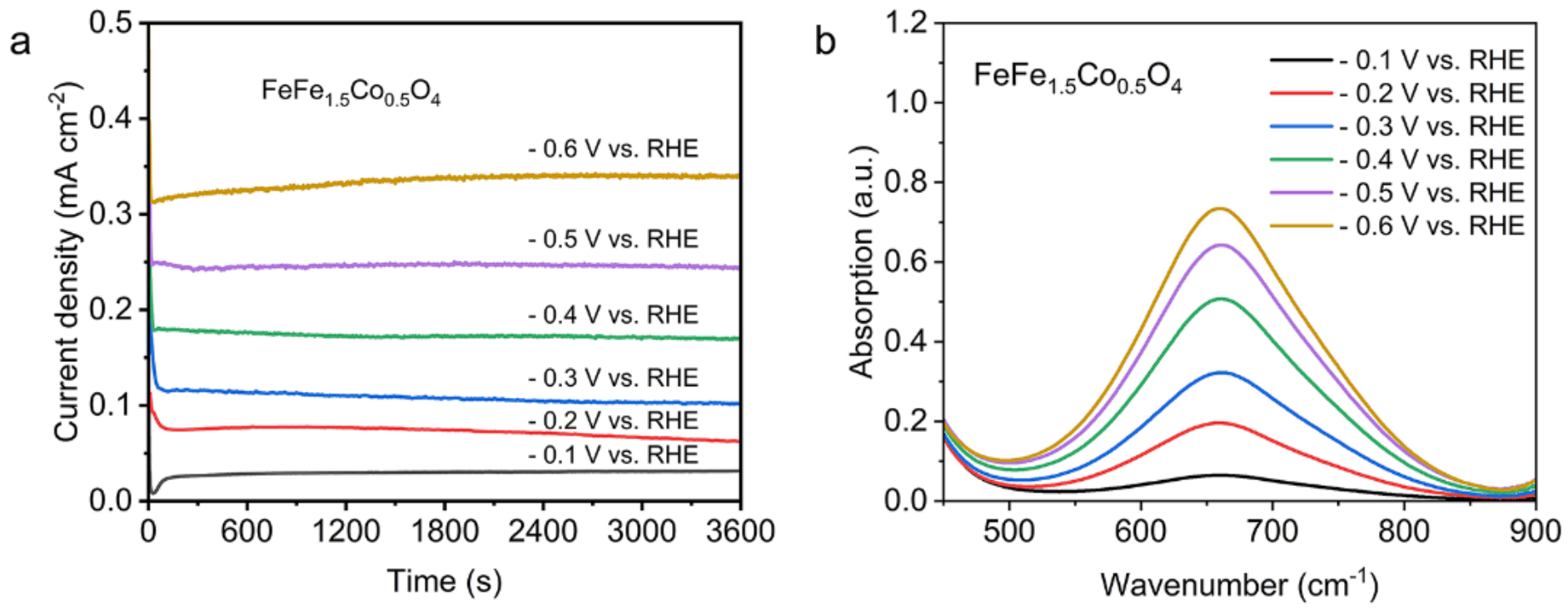

Figure S23. Quantification analysis of the generated ammonia after chronoamperometry measurements with different potentials in 1 M KOH + 0.1 M KNO_3 electrolyte over $FeFe_{1.5}Co_{0.5}O_4$. The current density curves of chronoamperometry measurements at different potentials (vs. RHE) over $FeFe_{1.5}Co_{0.5}O_4$ (a) and the corresponding UV-vis absorption spectroscopy of indophenol blue spectrophotometry. **and (c) $FeFeCoO_4$**

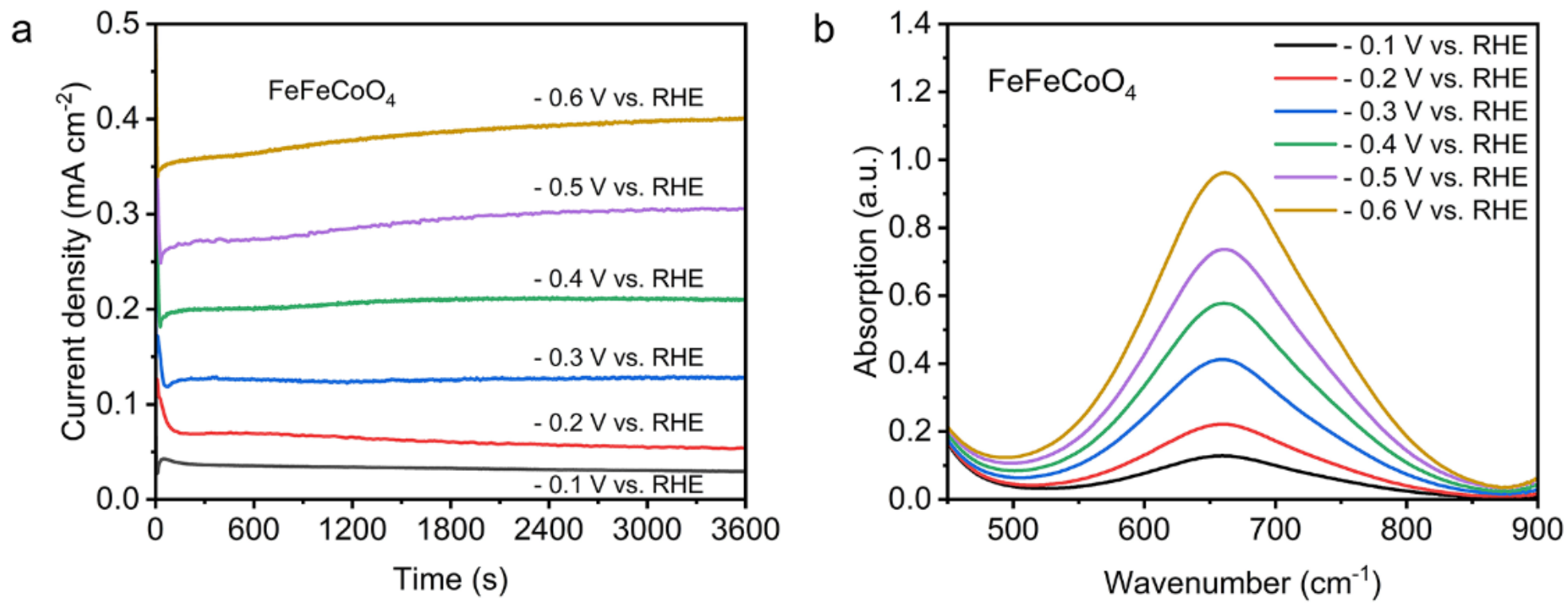

Figure S24. Quantification analysis of the generated ammonia after chronoamperometry measurements with different potentials in 1 M KOH + 0.1 M KNO_3 electrolyte over $FeFeCoO_4$. The current density curves of chronoamperometry measurements at different potentials (vs. RHE) over $FeFeCoO_4$ (a) and the corresponding UV-vis absorption spectroscopy of indophenol blue spectrophotometry.

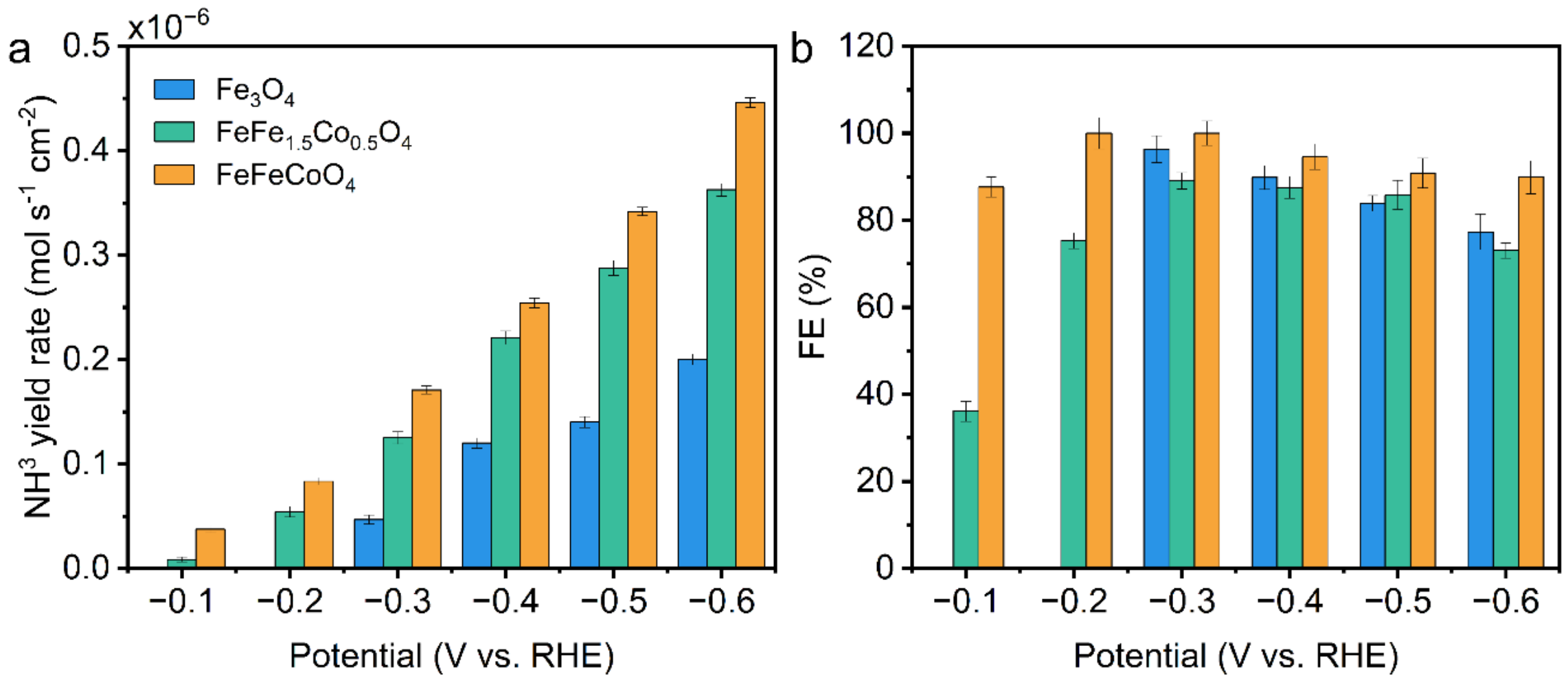

Figure S25. NO$_3$RR performance of Co$_x$Fe$_{3-x}$O$_4$. NH$_3$ yield rate (e), faradic efficiency (f) and energy efficiency (g) of Co$_x$Fe$_{3-x}$O$_4$ under different applied potentials in 1 M KOH + 0.1 M KNO$_3$ electrolyte.

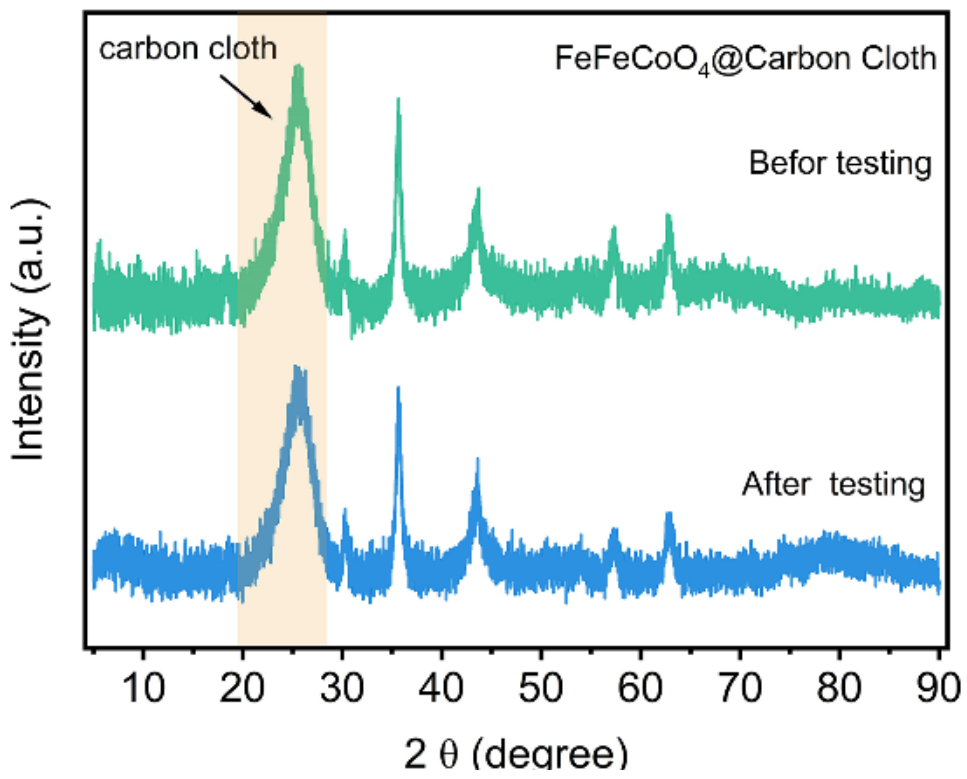

Figure S26. Structural stability investigation of $FeFeCoO_4$. XRD patterns of the $FeFeCoO_4$@carbon cloth before and after chronoamperometry measurement at -0.4 V vs RHE in 1 M KOH + 0.1 M KNO_3 electrolyte for 20 h. There is no obvious secondary phase, indicating the good structural stability of $FeFeCoO_4$.

Table S1. Comparison of NO_3RR performance of catalysts

No.	Materials	YNH3, mol s-1 cm-2	FE/%	E/V vs RHE	EE/%	References
**1**	$Ru_{15}Co_{85}$	5.40E-07	95	-0.5	35.1	Nat Catal 6, 402–414 (2023).
**2**	O-Cu-PTCDA	6.90E-09	85.9	-0.4	33.7	Nat. Energy 5, 605–613 (2020)
**3**	Ru-CuNW,	1.20E-06	93	-0.135	43.6	Nat. Nanotechnol. 17, 759–767 (2022).
**4**	Cu_2O	3.60E-09	74	-0.6	25.9	Nat. Mater. 24, 762–769 (2025).
**5**	$RuIn_3/C$	5.40E-08	97.6	-0.1	47.0	Nat Commun 14, 7368 (2023).
**6**	hcp-RuCo,	1.90E-07	95	-0.1	45.7	Nat Commun 16, 5742 (2025).
**7**	2D-Ru/NC,	7.40E-07	90	-0.9	27.0	Nat Commun 15, 10877 (2024).
**8**	FeTeSe,	6.90E-08	72	-0.75	23.3	Nat Commun 16, 3595 (2025).
**9**	OD-Cu,	9.70E-08	99.7	-0.6	34.9	Nat Commun 16, 3479 (2025).
**10**	M-Cu/Cu_2O	6.90E-08	95	-0.2	42.5	Nat Commun 16, 2392 (2025).
**11**	BECOF/PdCu	6.10E-07	91	-1.3	23.0	Nat Commun 16, 3717 (2025).
**12**	FL-Ag/HEA	8.10E-08	92.7	-0.57	33.0	Nat Commun 15, 9020 (2024).
**13**	FePc/TiO_2	2.80E-07	70	-0.8	22.1	Nat Commun 14, 8036 (2023).
**14**	M-N-C, FeN_4	1.00E-08	75	-0.8	23.6	Nat Commun 14, 4554 (2023).
**15**	Ag-MoS_2,	2.90E-07	88	-0.8	27.7	Nat Commun 16, 5715 (2025).
**16**	RuN_2-CuN_3	5.10E-08	95.7	-0.4	37.6	Nat Commun 16, 2167 (2025).
**17**	Co_6Ni_4	1.50E-06	99.2	-0.476	37.2	Nat Commun 16, 6161 (2025).
**18**	TiH1.97	1.40E-06	99.11	-0.7	32.9	Nat Commun 15, 9499 (2024).
**19**	G-RuCo	1.50E-06	93	-0.136	43.6	Nat Commun 15, 6278 (2024).
**20**	V-Cu NAE	1.30E-07	96	−0.3	40.2	Nat Commun 15, 2816 (2024).
**21**	Co–B/$Ru1_2$	2.50E-07	88	-0.2	39.4	Nat Commun 15, 8583 (2024).
**22**	Co_3O_4/Cu1-N-C	1.50E-06	94	-0.8	29.6	Nat Commun 15, 3619 (2024).
**23**	Fe-HESA NCs	1.30E-06	93.4	−0.6	32.7	Nat Commun 15, 6932 (2024).
**24**	Fe single atom catalyst,	1.30E-07	70	-0.85	21.5	Nat Commun 12, 2870 (2021).
**25**	dual atom FeCu-HNG	7.20E-08	61	-0.5	22.6	Nat Commun 14, 3634 (2023).
**26**	MgCoNiCuZn	4.40E-07	97.2	-0.4	38.2	Nat Commun 15, 260 (2024).
**27**	Cu@hNCNC	1.20E-07	90	-0.6	31.5	Sci. Adv. 10, eadm9325 (2024)
**28**	Cu26·4PF6	2.40E-08	85.1	-0.85	26.2	Sci. Adv. 10, eadn7556 (2024)
**29**	Ru─$Ni(OH)_2$	3.70E-07	96	-0.3	40.2	Adv. Mater. 2025, 37, 2417696
**30**	p-CN-CusLan–m	6.14E-07	97.7	-0.45	37.2	Adv. Mater. 2025, 37, 2415632
**31**	IrNiCu@Cu-20	3.80E-07	86	-0.1	41.4	Adv. Mater. 2024, 36, 2407889
**32**	Cu_6Sn_5-type HEI	1.50E-07	97.1	-0.3	40.6	Adv. Mater. 2025, 2501886

**33**	MnFeCoNiCu-HEA	1.70E-08	94.5	-0.6	33.0	Adv. Mater. 2025, 37, 2415739
**34**	CCZ-IOs	1.70E-07	92.1	-0.56	32.9	Adv. Mater. 2025, 2510680
**35**	Ru 3% -HEO	4.70E-08	91.3	-0.5	33.8	Adv. Mater. 2025, 2500224
**36**	CoN_4 -pyrr	5.40E-07	99.5	-0.8	31.4	Adv. Mater. 2025, 37, 2418681
**37**	**$FeFeCoO_4$**	**1.89E-06**	**97**	**-0.8**	**30.5**	**This work**

Table S2. The BET-normalized current density and yield rate for the three samples.

Catalyst	BET (m^2/g)	$A_{BET, loaded}$ (cm^2),	$J_{geo, -0.8V vs RHE}$ ($mA\ cm^{-2}$)	J_A ($mA\ cm^{-2}$)	$Y_{geo, -0.8 V vs RHE}$ ($mol^{-1}s^{-1}cm^{-2}$)	Y_A ($mol^{-1}s^{-1}cm^{-2}$)
**Fe_3O_4**	36.142	1807.1	623	0.345	6.94 x 10^{-7}	3.84 x 10^{-10}
**$FeFe_{1.5}Co_{0.5}O_4$**	15.636	781.8	916	1.172	1.33 x 10^{-6}	1.70 x 10^{-9}
**$FeFeCoO_4$**	14.781	739.05	1193	1.614	1.89 x 10^{-6}	2.56 x 10^{-9}

Table S3. The ECSA-normalized current density and NH_3 yield rate of the three samples (Fe_3O_4, $FeFe_{1.5}Co_{0.5}O_4$, and $FeFeCoO_4$) at -0.8 V vs RHE under 1M KOH +1 M KNO_3 electrolyte.

**Catalyst**	**ECSA** (**cm^2**)	**$J_{geo, -0.8 V vs RHE}$** (**mA cm^{-2}**)	**J_{ECSA}** (**mA cm^{-2}**)	**$Y_{geo,-0.8 V vs RHE}$** (**$mol^{-1}s^{-1}cm^{-2}$**)	**Y_{ECSA}** (**$mol^{-1}s^{-1}cm^{-2}$**)
**Fe_3O_4**	77.5	623	8.04	6.94×10^{-7}	8.95×10^{-9}
**$FeFe_{1.5}Co_{0.5}O_4$**	147.5	916	6.21	1.33×10^{-6}	9.02×10^{-9}
**$FeFeCoO_4$**	172.5	1193	6.92	1.89×10^{-6}	1.10×10^{-8}

References

[1] J. Hafner, *J. Comput. Chem.* **2008**, 29, 2044.

[2] K. P. McKenna, F. Hofer, D. Gilks, V. K. Lazarov, C. Chen, Z. Wang, Y. Ikuhara, *Nat. Commun.* **2014**, 5, 5740.

[3] E. Skúlason, V. Tripkovic, M. E. Björketun, S. Gudmundsdóttir, G. Karlberg, J. Rossmeisl, T. Bligaard, H. Jónsson, J. K. Nørskov, *J. Phys. Chem. C* **2010**, 114, 18182.
